# Transcriptional Profiling of SSEA‐1^+^ Endometrial Epithelial Progenitor Cells Highlights Their Role in Endometrial Regeneration, Remodeling, and Homeostasis

**DOI:** 10.1096/fj.202402861R

**Published:** 2025-04-29

**Authors:** Hannan Al‐Lamee, Jamie Soul, Daniel Green, Josephine Drury, Christopher J. Hill, Olga Vasieva, Anthony Valentijn, Alison Maclean, Andrew Drakeley, Nicola Tempest, Dharani K. Hapangama

**Affiliations:** ^1^ Department of Women's and Children's Health Institute of Life Course and Medical Sciences, Centre for Women's Health Research, University of Liverpool, Member of Liverpool Health Partners Liverpool UK; ^2^ Hewitt Centre for Reproductive Medicine Liverpool Women's NHS Foundation Trust Liverpool UK; ^3^ Liverpool Women's NHS Foundation Trust, Member of Liverpool Health Partners Liverpool UK; ^4^ Imperial College Healthcare NHS Trust London UK; ^5^ Computational Biology Facility University of Liverpool Liverpool UK; ^6^ Institute of Systems, Molecular and Integrative Biology University of Liverpool Liverpool UK; ^7^ Department of Pharmacology and Therapeutics, Institute of Systems, Molecular and Integrative Biology, Centre of Excellence in Long‐Acting Therapeutics (CELT) University of Liverpool Liverpool UK

**Keywords:** endometrial epithelium, endometrial stem/progenitor cells, endometrium, regeneration, SSEA‐1

## Abstract

Stage‐specific embryonic antigen‐1 (SSEA‐1)^+^ endometrial epithelial cells (EECs) assume the postulated stem/progenitor cell niche within the human endometrium. Previous studies have demonstrated that isolated SSEA‐1^+^ cells have a higher capacity to generate organoids in a three‐dimensional matrix, a lower steroid hormone receptor expression, and higher telomerase activity with longer telomere lengths. Here, we present the transcriptomic profile of isolated SSEA‐1^+^ EECs from eight endometrial biopsies compared to SSEA‐1^−^ EECs. Transcriptome and pathway analysis indicate that SSEA‐1^+^ EECs play an important role in endometrial regeneration, remodeling and neovascularization as expected from a basal progenitor population. We show that SSEA‐1^+^ EECs play a role in endometrial tissue homeostasis and tumor suppression, and bioinformatically identify potential upstream regulators such as SPDEF and TGFB1, which may be involved in these mechanisms. In vitro EEC organoid models also demonstrate SSEA‐1^+^ EECs to exhibit estrogen responsive proliferation evidenced by stronger immunostaining for progesterone receptor and Ki‐67. Our data further suggest a more quiescent, less hormone responsive phenotype for SSEA‐1^+^ EECs that co‐localize to *SOX9* ^+^ EECs within in silico analysis, thus validating previous studies.

## Graphical abstract

Sampling, isolation, cell sorting and microarray analysis of SSEA‐1^+^ endometrial epithelial progenitor cells. Gene expression data was analysed using computational pathway analysis methods and internally validated using real‐time quantitative PCR and externally validated using dual‐label immunofluorescence and endometrial epithelial organoid models.

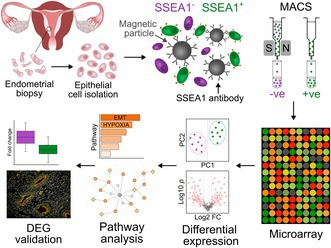

## 1 Introduction

The human endometrium is an indispensable organ in the female reproductive system, with an unparalleled regenerative ability. It is organized into two functionally and phenotypically different layers: (1) a superficial functionalis, which appears only during the reproductive years of a woman's life, to undergo an iterative monthly cycle of shedding, regeneration, remodeling, and transformation according to the ovarian steroid cue, and (2) a permanent deeper basalis, which persists throughout a woman's life. Endometrial stem/progenitor cells (SPCs) are postulated to reside in the deeper basalis and hypothesized to be responsible for the extraordinary capacity for full phenotypic and functional restoration of the endometrial functionalis layer [1, 2]. This efficient and scarless regeneration of the endometrial functionalis happens after menstrual shedding, parturition, and in the revived post‐menopausal endometrium, when stimulated with exogenous hormones [2, 3, 4, 5, 6, 7]. Several types of SPCs have been identified within the endometrium, including endometrial mesenchymal stem cells (eMSCs), endometrial epithelial progenitor cells (eEPCs), and side population cells [8].

In 2013, we described an endometrial epithelial cell (EEC) sub‐population that express surface marker stage‐specific embryonic antigen‐1 (SSEA‐1). SSEA‐1^+^ EECs assume the postulated SPC location, the basalis, while demonstrating some expected SPC functional properties. SSEA‐1^+^ EECs exhibit higher telomerase activity, longer telomere lengths, quiescence, and resistance to hormonal stimuli (lower estrogen receptor α (*ESR1*) and progesterone receptor (*PR*)), suggesting a less differentiated phenotype compared to SSEA‐1^−^ EECs [2, 8, 9]. In an in vitro three‐dimensional (3D) culture system, SSEA‐1^+^ EECs demonstrated a higher propensity to generate endometrial gland‐like spheroids and growth similar to that observed after endometrial denudation [2]. SSEA‐1^+^ EECs also express nuclear SRY‐box transcription factor 9 (nSOX9) and nuclear β‐catenin, suggesting activated Wnt signaling [2]. The involvement of SSEA‐1^+^/nSOX9^+^ EECs in endometrial pathologies has been implied by their aberrant location in women with endometriosis [10]. Subsequent studies have proposed other EEC progenitor markers including leucine‐rich repeat containing G protein‐coupled receptor 5 (*LGR5*) [11], axis inhibition protein 2 (*AXIN2*) [12] and cadherin 2 (*CDH2*) [13], with a suggested epithelial SPC hierarchy based on the exact location within the basalis glands [13].

Since the EEC SPC population makes a vital contribution to routine endometrial regeneration and pregnancy establishment, understanding the transcriptional profile and the associated functional pathways of these cells is essential to understanding their role within normal endometrial physiology [8, 9]. Furthermore, such data will be invaluable for the identification of pathology‐related aberrations in SPC populations. In this study, we characterize the transcriptional profile of isolated SSEA‐1 enriched (SSEA‐1^+^) EECs compared to SSEA‐1 depleted (SSEA‐1^−^) EECs and identify differences in key functional pathways. Functional work employing an in vitro endometrial organoid model demonstrated hormonal regulation in endometrial regeneration and differentiation and the role SSEA‐1^+^ EECs play in this process.

## 2 Materials and Methods

### 2.1 Transcriptome Analysis of EECs Enriched for SSEA‐1^+^

Raw microarray data is available from the Gene Expression Omnibus (GEO at the National Center for Biotechnology Information, http://www.ncbi.nlm.nih.gov/geo/; accession number GSE280323).

### 2.2 Human Endometrial Sample Collection

Endometrial samples were collected with informed, written consent from women attending the Liverpool Women's Hospital for benign gynecological conditions. For microarray and RT‐qPCR experiments, endometrial tissue samples were obtained from eight patients. Three further endometrial samples were obtained for epithelial organoid culture. All patients had regular menstrual cycles and did not take any hormonal therapy in the 3 months prior to enrollment. Age of participants ranged between 30 and 47 years. Further clinical characteristics of the participants are detailed in Table S1. Collection of human endometrial samples was approved by the Adult Ethics committees (REC references; 09/HI005/55, 19/WA/0271 and 19/SC/0449). From those attending for a hysterectomy, a full thickness wedge endometrial biopsy was taken, while a pipelle endometrial sample was obtained from those attending for a laparoscopy.

### 2.3 Isolation of Endometrial Epithelial Cells and Epithelial Cell Sorting

Freshly harvested endometrial tissue was mechanically and enzymatically digested and filtered into stromal and epithelial cell fractions [2, 10]. The epithelial single‐cell suspension was labeled with anti‐SSEA1 (CD15) MicroBeads (#130‐094‐530, Miltenyi Biotec, UK) and magnetic bead (MACS) sorted into SSEA‐1^+^ and SSEA‐1^−^ populations using MACS separation columns (MS columns, Miltenyi Biotec, UK) according to the manufacturer's instructions. Cell purity using this technique has previously been reported [2].

### 2.4 RNA Extraction and cDNA Synthesis

Total RNA from SSEA‐1^+^ and SSEA‐1^−^ fractions was extracted using the TRIzol Plus RNA Purification System (Invitrogen Life Technologies, Paisley, UK) and quantified using a NanoDrop 1000 (ThermoFisher Scientific, UK) and was DNAse treated (Promega, UK) to remove genomic DNA according to the manufacturer's instructions. A portion of DNAse treated RNA was retained for microarray analysis from each sample before the remaining RNA was used for cDNA synthesis. 1 μg of DNase treated RNA was reverse transcribed using iScript cDNA Synthesis Kit (Bio‐Rad Laboratories Ltd., UK) as per the manufacturer's instructions.

### 2.5 Microarray Analysis

Microarray analysis was performed at the Centre for Genomic Research (University of Liverpool). DNase treated RNA was quantified and the quality assessed using the Agilent 2100 Bioanalyser RNA 6000 Nano and Pico chips (Agilent Technologies, Santa Clara, US). Samples were prepared for hybridization onto Agilent SurePrint G3 format 8 × 60k Human Genome Array (Agilent Technologies). 40 ng RNA was used in the initial input and the Agilent Low Input Quick Amp Labelling Kit (Agilent Technologies) was used for target preparation, using the Two‐Color Microarray‐Based Gene Expression Analysis protocol, according to the manufacturer's instructions. Labeled amplified cRNA was purified using the Qiagen RNeasy Minikit (Qiagen, Hilden, Germany), according to the manufacturer's instructions. The cRNA was quantified by Nanodrop and the yield and specific activity calculated. A total of 600 ng (300 ng of both Cy3 and Cy5) of labeled cRNA was fragmented and, following the addition of 2 × HiRPM hybridization buffer, samples were loaded onto arrays and hybridized for 17 h at 65°C in an Agilent hybridization oven (Agilent Technologies). Following hybridization, the arrays were washed using the Agilent gene expression wash buffer kit and scanned using the Agilent G2505C scanner with Agilent G3_GX_2colour settings (Agilent Technologies). Data was extracted using the Agilent feature extraction software version 11.0.1.1.

Data processing and statistical analysis of microarray data was performed using R (Version 4.2.3) (R Core Team, 2024. R: A language and environment for statistical computing. Vienna: R Foundation for Statistical Computing. Available at: https://www.r‐project.org/). Data was background corrected using the “normexp” function [14], normalized within arrays using “vsn” [15] and “loess” [16] methods, and normalized between arrays using the “Aquantile” method [17] in limma. Log2 normalized expression data was centred and visualized with principal component analysis. Microarray data was analyzed in limma by applying a linear model using the empirical bayes moderated *t* test, and *p*‐values were adjusted to correct for multiple tests using false discovery rate (FDR) using the Benjamini and Hochberg (BH) method [18]. Differentially expressed genes (DEGs) were filtered using FDR < 0.05 and logFC > 1 or < −1.

### 2.6 Pathway Enrichment Analysis

Enriched Hallmark, Gene Ontology (GO) and Kyoto Encyclopedia of Genes and Genomes (KEGG) pathway analysis was performed using the Over Representation Analysis method [19] via the ClusterProfiler “enricher” function [20] on the DEGs using all genes inputted into the differential expression analysis as the background. Multiple testing correction was performed using BH, and a FDR cutoff of 0.05 was applied.

### 2.7 Ingenuity Pathway Analysis

Canonical pathway analysis, upstream regulators, mechanistic and causal network analysis, and associations with diseases and functions were generated using QIAGEN Ingenuity Pathway Analysis (IPA) (QIAGEN Inc. https://digitalinsights.qiagen.com/IPA) [21] by inputting the DEGs into the “core analysis” function and a cut‐off set to logFC < −1 and > 1. The significance of biofunctions and canonical pathways was tested by the Fisher's Exact Test *p*‐values and analysis cutoffs set to *p*‐value < 0.05 and *z*‐score > 2 (to assess activation) or < −2 (to assess inhibition).

### 2.8 Comparison With Publicly Available Data

External validation of microarray data against publicly available endometrial single cell spatial transcriptomic data published by Garcia‐Alonso et al. [22] was performed using R. Garcia‐Alonso et al. pre‐processed single cell data was visualized using the previously reported UMAP coordinates and cell type annotations [23]. To assess epithelial cell type distribution for expression of matrix metallopeptidase 7 (MMP7) and matrix metallopeptidase 26 (MMP26) a normalized log2 expression level > 2 was set.

### 2.9 Quantitative Real‐Time Polymerase Chain Reaction (RT‐qPCR)

1 μL cDNA was amplified in triplicate for 40 cycles in a final reaction volume of 10 μL using iTaq Universal SYBR Green Supermix (Bio‐Rad Laboratories Ltd., Hemel Hempstead, Hertfordshire, UK) and a Biorad CFX Connect Real‐Time System (Bio‐Rad Laboratories Ltd.). No template and no reverse transcriptase controls were included for each target in each experiment. Relative gene expression was calculated and normalized to the reference genes glyceraldehyde 3‐phosphate dehydrogenase (*GAPDH*), tyrosine 3‐monooxygenase/tryptophan 5‐monooxygenase activation protein zeta (*YWHAZ*) and actin beta (*ACTB*) and normalized to Ishikawa endometrial cancer cell line (ISK) as an internal control using Biorad CFX manager (version 3.1, Bio‐Rad Laboratories Ltd). The amplification products were verified using gel electrophoresis. The PCR primers are detailed in Table S2.

### 2.10 Immunofluorescence

Dual immunofluorescence (IF) was performed on 3 μm formalin‐fixed paraffin‐embedded (FFPE) tissue sections. Primary anti‐rabbit MMP7, MMP26, cluster differentiation 47 (CD47) or chromosome 11 open reading frame 52 (C11orf52) antibodies were applied to sections alongside Alexa Fluor 488 conjugated SSEA1 and incubated at 4°C overnight. A combination of anti‐mouse IgG and anti‐rabbit IgG was used as a negative control. The secondary antibody anti‐rabbit Alexa Fluor 555 (Cell Signaling Technology, Hitchin, UK) was used at the recommended concentration. Details of antigen‐retrieval conditions and antibody concentrations are listed in Table S3. To diminish unwanted background autofluorescence, slides were incubated at room temperature with TrueVIEW autofluorescence quenching kit (Vector Laboratories, Peterborough, UK) as per the manufacturers instructions, before mounting in Vectashield with DAPI (Vector Laboratories). IF‐stained slides were visualized on a Zeiss LSM 800 confocal microscope fitted with GaAsP detectors. Laser lines used were of 405, 488, and 561 nm excitation wavelengths. Zen Blue software was used for image capture and Image J [24] for processing.

### 2.11 Endometrial Epithelial Organoid Culture

Endometrial tissue was minced into small pieces (< 1 mm) using a scalpel blade and digested with 1 mg/mL Dispase II (Gibco), 2 mg/mL collagenase type I (Gibco) and 80  μg/ml deoxyribonuclease (DNase) 1 (Merck) for ~1 h at 37°C in a shaking water bath. Digests were periodically triturated to enhance tissue breakdown and observed under a microscope to check for the presence of free epithelial glands. The digests were passed through a 40 μm cell sieve (Falcon) to separate glandular (retentate) and stromal (flow‐through) elements. Glandular elements were collected by backwashing the sieve into a collection dish. Endometrial epithelial organoids were generated from the glandular fraction following an established protocol [25]. Briefly, glands were centrifuged at 500x *g* for 5 min and resuspended in phenol‐free DMEM/F12 (Gibco). Glands were partially fragmented by trituration and centrifuged at 500x *g* for 5 min. The supernatant was removed and glands resuspended in ice‐cold phenol‐free Matrigel (Corning) at a 1:20 ratio (glands: Matrigel). The gland‐Matrigel mixture was deposited as 20 μL droplets in 48 well tissue culture plates and allowed to set at 37°C for 15 min. Droplets were overlaid with expansion medium (Table S4) and maintained at 37°C under 5% CO_2_. Organoids were cultured to passage 2/3 before hormone treatment; organoids were passaged and allowed to establish for 48 h. Media was supplemented with 10 nM β‐oestradiol (E2, Sigma Aldrich) for 24 h, followed by 10 nM E2, 1 μM medroxyprogesterone acetate (MPA, Sigma Aldrich) and 500 μM cyclic adenosine monophosphate (cAMP, Sigma Aldrich) for 5 days. Organoids were collected using Cell Recovery Solution (Corning) and fixed with 10% neutral buffered formalin for 1 h. Fixed organoids were embedded in HistoGel (Fisher Scientific) and embedded in paraffin.

### 2.12 Organoid Immunohistochemistry

Immunohistochemistry (IHC) was performed as previously described [2]. FFPE organoids were sectioned at 3 μm, dewaxed, and rehydrated prior to heat‐induced epitope retrieval in a pressure cooker. Endogenous peroxidase activity was blocked with 0.3% hydrogen peroxide (Thermo Fisher Scientific, Runcorn, UK) for 10 min, prior to incubation with diluted primary antibody overnight at 4°C (Table S5). The appropriate ImmPRESS polymer‐based system was applied for 30 min at room temperature, and visualization was with ImmPACT DAB, following the manufacturer's instructions (Vector Laboratories). Sections were counterstained using Gill II Hematoxylin (Thermo Fisher Scientific), dehydrated, cleared, and mounted using Consul‐Mount (Thermo Fisher Scientific). Matching isotype IgG antibodies replaced the primary antibody as a negative control, with an internal positive control in each staining run. Slides were digitalized using an Aperio CS2 slide scanner (Leica Biosystems, Milton Keynes, UK). Scoring was assessed by counting the proportion of positively stained organoids for the primary antibody versus the proportion of negatively stained organoids.

### 2.13 Statistical Analysis

GraphPad Prism software (version 9.0, GraphPad Software, San Diego, CA, USA) was used to statistically analyze paired qPCR data, using the Wilcoxon matched‐paired signed rank test and the level of statistical significance set at *p*‐value < 0.05.

## 3 Results

### 3.1 SSEA‐1^+^ Epithelial Cells have a Distinct Transcriptional Profile Compared with other Epithelial Cells within the Human Endometrium

The differential functional ability of EEC subtypes is demonstrated by the well‐established region‐specific functional differences in the endometrium (e.g., functionalis, the site for embryo‐implantation, and basalis, the site retaining regenerative capacity), and SSEA‐1^+^ EECs have demonstrated some functional properties in keeping with SPCs [2, 10]. Therefore, we sought to examine the anticipated distinct transcriptome between SSEA‐1^+^ and the more differentiated SSEA‐1^−^ EEC population using freshly isolated and sorted cells from eight human endometrial biopsies. Transcriptomic analysis of the SSEA‐1^+^ versus SSEA‐1^−^ EEC populations was performed using a two‐color microarray‐based gene expression technology.

**FIGURE 1:**
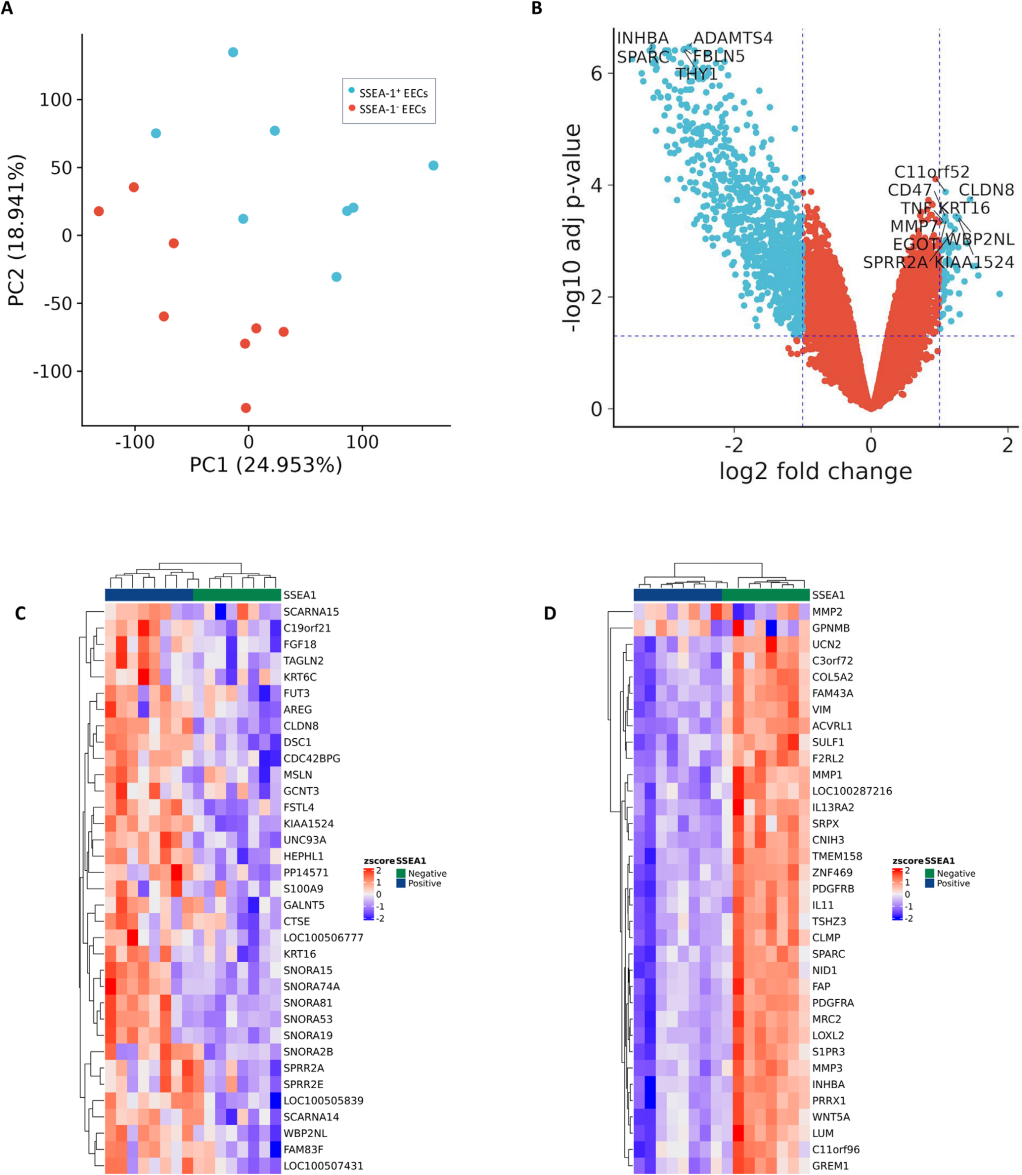
(A) Principal component analysis (PCA) plot of SSEA‐1^+^ EECs (blue) and SSEA‐1^−^ EECs (red) across all eight endometrial samples showing distinct clustering. (B) Volcano plot of all 22 860 expressed genes. Blue points represent differentially expressed genes (DEGs) (FDR < 0.05) (71 upregulated DEGs with logFC > 1 and 983 downregulated DEGs with logFC < −1). Top upregulated and downregulated DEGs have been annotated within the volcano plot. (C) Heat map and hierarchical clustering of the mRNA expression of top 35 upregulated DEGs, showing distinct transcriptional clustering between SSEA‐1^+^ EECs (left) and SSEA‐1^−^ EECs (right). (D) Heat map and hierarchical clustering heatmap of the mRNA expression of top 35 downregulated DEGs, showing distinct transcriptional clustering between SSEA‐1^+^ EECs (left) and SSEA‐1^−^ EECs (right).

Principal component analysis (PCA) demonstrated distinct clustering of SSEA‐1^+^ and SSEA‐1^−^ EECs (Figure 1A). A total of 1054 (71 upregulated and 983 downregulated) differentially expressed genes (DEGs) were identified in the SSEA‐1^+^ EECs compared to the SSEA‐1^−^ EEC population (Figure 1B and Table 1). Hierarchical clustering was observed for SSEA‐1^+^ and SSEA‐1^−^ EEC populations based on their DEGs (Figure 1C,D).

**TABLE 1:** List of differentially expressed genes (DEGs) in the SSEA‐1^+^ versus SSEA‐1^−^ endometrial epithelial cell populations (FDR < 0.05). 71 upregulated genes with logFC > 1 and top 100 downregulated genes with logFC < −1.

DEGs	Gene
Upregulated genes	*SNORA15, SNORA74A, SNORA19, SCARNA14, CLDN8, GCNT3, SNORA81, LOC100506777, SNORA53, WBP2NL, KIAA1524, GALNT5, KRT6C, TAGLN2, KRT16, SNORA2B, SPRR2A, DSC1, LOC100505839, CTSE, S100A9, FUT3, LOC100507431, FSTL4, PP14571, HEPHL1, SPRR2E, FGF18, SCARNA15, AREG, MSLN, C19orf21, UNC93A, FAM83F, CDC42BPG, EGOT, RHOD, LOC645638, C11orf52, HS3ST1, SNORA36B, LINC00485, CD47, IL18, FAM107A, SLC44A3, PGBD5, PRAME, MMP7, MGC34034, CAPN1, OR4N3P, SNORD17, MANSC1, TNF, GLYCAM1, ZNF214, SPINT1, DUOXA2, TRIM48, SCARNA8, DCTPP1, RASSF7, HOOK2, SCARNA4, LOC100506389, SCARNA16, RAB25, APOBEC3A, ELF3, CFTR*
Downregulated genes	*MMP3, WNT5A, NID1, PDGFRA, PDGFRB, SPARC, INHBA, LOXL2, FAP, MMP2, TMEM158, UCN2, CNIH3, C3orf72, LUM, COL5A2, CLMP, SULF1, F2RL2, MRC2, S1PR3, ZNF469, GPNMB, C11orf96, MMP1, GREM1, ACVRL1, VIM, SRPX, LOC100287216, FAM43A, TSHZ3, PRRX1, IL11, IL13RA2, CTSK, TBX2, FAM65C, TMEM200A, COL1A1, CDH11, TMEM45A, NEFM, COL7A1, C13orf33, TOX2, PAMR1, BMPER, COL13A1, MME, RUNX1T1, ZEB1, PLAT, HTRA1, A2M, BDKRB2, THY1, FOXL2, FBLN5, PPAPDC3, PCDH18, NDN, TSPAN5, DCN, COX7A1, TMEM204, PDE4B, CNN1, ANTXR1, DACT3, REN, ADAMTS4, SPON2, ARHGAP22, LEPREL2, PRKAR2B, DAB2, STRA6, ITGA4, KRT34, THBS2, STON1, TPM2, COL12A1, ADAMTS5, MSRB3, APCDD1, SYT7, ITGA1, MOXD1, SNORD114‐15, TBX3, LOC100507150, AEBP1, SPARCL1, STMN2, KDR, DSEL, DOCK4, AR*

### 3.2 Gene Enrichment Analysis Demonstrates that SSEA‐1^+^ EECs are Involved in Endometrial Regeneration, Adhesion, and Remodeling

#### 3.2.1 Gene Ontology Pathways

**FIGURE 2:**
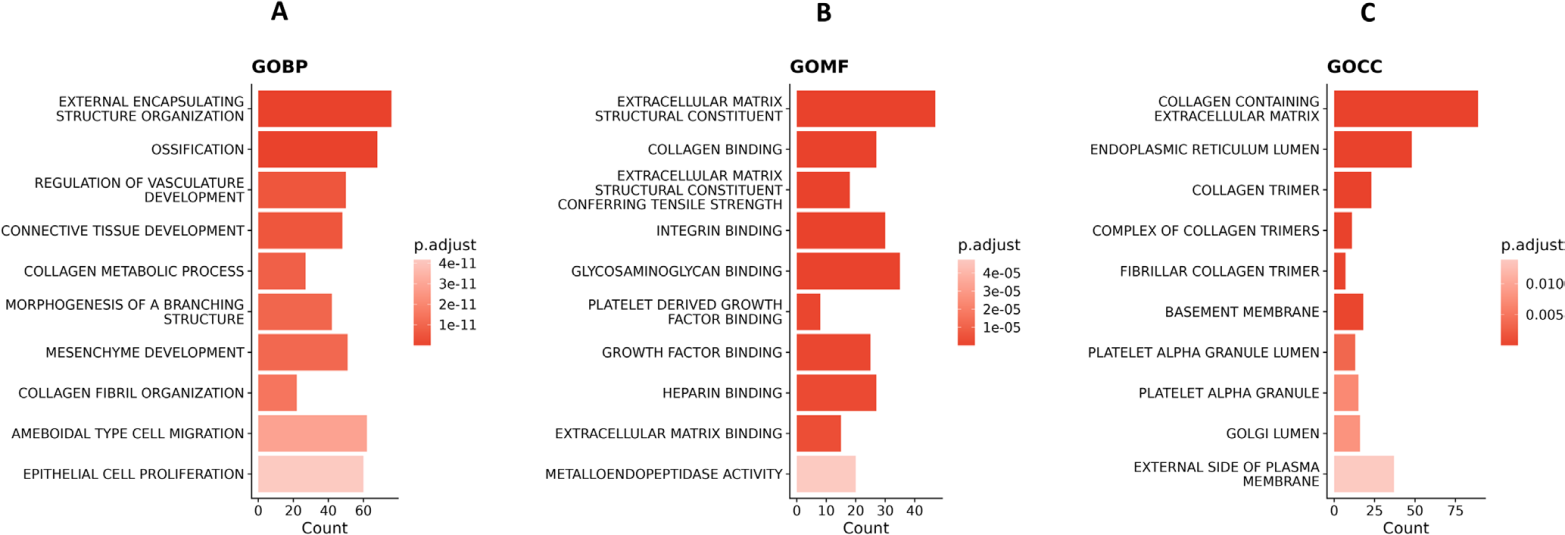
Gene enrichment analysis. (A) Bar chart of top 10 enriched Gene Ontology (GO)‐Biological Processes (BP). (B) Enrichment plot of top 10 enriched GO‐Molecular Functions (MF). (C) Bar chart of top 10 enriched GO‐Cellular Components (CC).

GO pathways were analyzed according to three functional groups: biological processes, molecular functions and cellular components. The most significantly enriched pathways were related to structural organization and extracellular matrix (ECM). The top three biological processes included “external encapsuling structure organization” (76 genes, FDR = 1.08e−27), “ossification” (68 genes, FDR = 6.39e−15) and “regulation of vasculature development” (50 genes, FDR = 5.26e−12) (Figure 2A and Table 2). The top three most significantly enriched molecular functions were “extracellular matrix structural constituent” (47 genes, FDR = 4.48e−19), “collagen binding” (27 genes, FDR = 5.27e−16), and “extracellular matrix structural constituent conferring tensile strength” (18 genes, FDR = 1.24e−10) (Figure 2B and Table 3). The top three most significantly enriched cellular components were “collagen containing extracellular matrix” (89 genes, FDR = 3.12e−26), “endoplasmic reticulum lumen” (48 genes, FDR = 8.01e−09), and “collagen trimer” (23 genes, FDR = 3.48e−08) (Figure 2C and Table 4).

**TABLE 2:** Top 10 most significantly enriched Gene Ontology (GO)‐Biological Processes (BP) pathways in the SSEA‐1^+^ versus SSEA‐1^−^ endometrial epithelial cell populations.

TOP 10 enriched GO‐BP pathways	FDR	Gene ratio	Enriched genes
External encapsulating structure organization	1.08e−27	76/820	*MMP7, TNF, SPINT1, ELF3, POSTN, TGFBI, FOXF2, MMP11, LAMB1, AGT, FLRT2, COL4A2, EXT1, MMP19, SERPINH1, TMEM38B, COL24A1, COL4A1, TIE1, ADAMTS3, ITGA8, COL23A1, CRISPLD2, COL5A3, EGFL6, WT1, MYH11, MMP14, HAS2, COL15A1, PRSS2, MMP16, ANGPTL7, RECK, EFEMP2, MMP9, FBLN2, LOX, FMOD, TGFB2, TNFRSF11B, FSCN1, MMP10, COL5A1, FKBP10, MMP12, ADAMTS10, BMP2, COL8A1, COL1A2, DDR2, COL3A1, MFAP4, FOXF1, PDPN, AEBP1, ADAMTS5, COL12A1, ADAMTS4, ANTXR1, FBLN5, COL13A1, COL1A1, CTSK, GREM1, MMP1, ZNF469, SULF1, COL5A2, LUM, MMP2, FAP, LOXL2, PDGFRA, NID1, MMP3*
Ossification	6.39e−15	68/820	*FGF18, AREG, TNF, FHL2, VCAN, DKK1, PPARGC1B, TWIST2, SNAI1, TMEM119, DCHS1, CTHRC1, GJA1, EXT1, TMEM38B, FGR, WNT4, CYP27B1, TNFAIP6, HGF, EGR2, CCR1, SRGN, CEBPB, RORB, RUNX2, ECM1, FAM20C, TWIST1, GLI1, XYLT1, PENK, GLI3, SMO, RASSF2, PTN, NPR2, STC1, MMP14, ENPP1, MMP16, ITGA11, CLEC11A, MGP, MMP9, LOX, COL6A1, TGFB2, PBX1, HAND2, SNAI2, SLC24A3, BMP2, VEGFC, COL1A2, DDR2, SOX11, IGF2, LEF1, COL13A1, CDH11, COL1A1, CTSK, GREM1, MRC2, COL5A2, MMP2, WNT5A*
Regulation of vasculature development	5.26e−12	50/820	*FGF18, TNF, EMP2, THBS1, PLXND1, AGT, PDE3B, SERPINF1, HOXA5, COL4A2, ADAM12, DLL1, GATA2, WNT4, HGF, HMGA2, FLT1, CLDN5, GPR4, TIE1, NINJ1, PGF, ECM1, TWIST1, VASH1, CCBE1, NPPB, ANGPTL7, CHI3L1, RECK, TGFB2, ISM1, VEGF, SEMA5A, IL1B, ADM, AKT3, ANGPT2, PRKCA, TEK, KDR, THBS2, DCN, BMPER, ACVRL1, GREM1, GPNMB, SULF1, SPARC, WNT5A*
Connective tissue development	5.26e−12	48/820	*FGF18, TGFBI, FOXD1, HOXA5, SNAI1, EXT1, ARID5B, SERPINH1, SOX6, HMGA2, BGN, PPARGC1A, GPR4, RUNX2, HOXA3, EFEMP1, ECM1, HOXD3, WT1, ACTA2, GLI3, SOX5, RARB, STC1, MAF, NAMPT, CHI3L1, MGP, LOX, WNT5B, CHST11, HAND2, COL5A1, ANXA6, SNAI2, BMP2, COL3A1, HOXA11, ZEB1, COL1A1, CTSK, PRRX1, ACVRL1, GREM1, SULF1, LOXL2, PDGFRB, WNT5A*
Collagen metabolic process	8.18e−12	27/820	*MMP7, MMP11, MMP19, SERPINH1, WNT4, ADAMTS3, LARP6, MMP14, PRSS2, MMP16, RCN3, MMP9, MMP10, COL5A1, MMP12, F2R, COL1A2, MFAP4, COL1A1, CTSK, VIM, MMP1, MRC2, MMP2, FAP, PDGFRB, MMP3*
Morphogenesis of a branching structure	1e−11	42/820	*AREG, TNF, SPINT, FOXD1, PLXND1, AGT, HOXA5, DCHS1, VDR, EXT1, WNT4, COL4A1, HGF, FGF7, TIE1, EDNRA, SALL1, WT1, GLI3, SMO, RSPO3, MMP14, WNT2, PKD2, NFATC4, PRDM1, LAMA1, PBX1, SNAI2, BMP2, ADM, LEF1, FOXF1, HOXA11, AR, KDR, TBX3, COL13A1, TBX2, GREM1, SULF1, WNT5A*
Mesenchyme development	1.08e−11	51/820	*FOXF2, FOXD1, KITLG, TGFB1I1, HOXA5, HEYL, SNAI1, DCHS1, CITED2, ZFPM2, EXT1, WNT4, HGF, HMGA2, EDNRA, TWIST1, RBM24, WT1, ACTA2, ROBO1, SMO, FGFR1, HAS2, WNT2, ACTG2, PKD2, VASN, RADIL, TGFB2, HAND2, ANXA6, SNAI2, BMP2, SEMA5A, IL1B, CDH2, SOX11, LEF1, FOXF1, HTR2B, PDPN, TBX3, DAB2, DACT3, COL1A1, TBX2, ACVRL1, GREM1, LOXL2, PDGFRB, WNT5A*
Collagen fibril organization	1.46e−11	22/820	*MMP11, EXT1, SERPINH1, ADAMTS3, COL5A3, EFEMP2, LOX, FMOD, TGFB2, COL5A1, FKBP10, COL1A2, DDR2, COL3A1, AEBP1, COL12A1, COL13A1, COL1A1, GREM1, COL5A2, LUM, LOXL2*
Ameboidal type cell migration	2.89e−11	62/820	*KRT16, FGF18, TNF, RAB25, RIN2, EMP2, THBS1, PTPRG, PLXND1, KITLG, AGT, SERPINF1, GJA1, ARID5B, GATA2, FGF7, FSTL1, DAAM2, KANK2, EDNRA, TWIST1, NR2F2, VASH1, ACTA2, PRSS3, ROBO1, SMO, CCBE1, PTN, FGFR1, STC1, HAS2, NANOS1, SYDE1, CDH13, MMP9, ZEB2, RADIL, TGFB2, HAND2, ANXA6, VEGFC, SEMA5A, DDR2, CDH2, APOE, SRPX2, AKT3, ANGPT2, HTR2B, PRKCA, TEK, KDR, ITGA4, DCN, BMPER, ACVRL1, GREM1, FAP, LOXL2, SPARC, WNT5A*
Epithelial cell proliferation	4.16e−11	60/820	*FGF18, AREG, TNF, THBS1, MTSS1, LAMB1, SERPINF1, HOXA5, VDR, GATA2, HGF, FGF7, LIMS2, FLT1, CEBPB, TIE1, PGF, ECM1, TWIST1, KLF9, NR2F2, GLI1, VASH1, ROBO1, NLRC3, SMO, PTN, CCL26, FGFR1, MMP14, HAS2, WNT2, CDH13, TGFB2, SNAI2, MMP12, BMP2, VEGFC, SEMA5A, COL8A1, APOE, SOX11, IGF2, AKT3, HTR2B, PRKCA, TEK, AR, KDR, ITGA4, DAB2, HTRA1, BMPER, TBX2, ACVRL1, SULF1, FAP, LOXL2, SPARC, WNT5A*

**TABLE 3:** Top 10 most significantly enriched Gene Ontology (GO)‐Molecular Functions (MF) pathways in the SSEA‐1^+^ versus SSEA‐1^−^ endometrial epithelial cell populations.

Top 10 enriched GO‐MF pathways	FDR	Gene ratio	Enriched genes
Extracellular matrix structural constituent	4.48e−19	47/737	*POSTN, TGFBI, VCAN, THBS1, LAMB1, COL4A2, CTHRC1, COL24A1, COL4A1, BGN, EFEMP1, ECM1, COL23A1, COL5A3, COL6A2, EDIL3, COL15A1, VWF, CHI3L1, EFEMP2, MGP, FBLN2, COL6A1, FMOD, LAMA1, LTBP2, COL6A3, COL5A1, PCOLCE, COL8A1, COL1A2, COL3A1, SRPX2, MFAP4, AEBP1, COL12A1, THBS2, DCN, FBLN5, COL13A1, COL7A1, COL1A1, SRPX, COL5A2, LUM, SPARC, NID1*
Collagen binding	5.72e−16	27/737	*TGFBI, THBS1, SERPINH1, C1QTNF1, ITGA10, COL5A3, COL6A2, CCBE1, LRRC15, VWF, ITGA11, MMP9, LOX, COL6A1, PCOLCE, MMP12, DDR2, SPARCL1, AEBP1, ITGA1, ANTXR1, DCN, CTSK, MRC2, LUM, SPARC, NID1*
Extracellular matrix structural constituent conferring tensile strength	1.24e−10	18/737	*COL4A2, COL24A1, COL4A1, COL23A1, COL5A3, COL6A2, COL15A1, COL6A1, COL6A3, COL5A1, COL8A1, COL1A2, COL3A1, COL12A1, COL13A1, COL7A1, COL1A1, COL5A2*
Integrin binding	2.54e−08	30/737	*TGFBI, EMP2, THBS1, ITGA7, LAMB1, FCER2, ITGA10, ITGA8, ESM1, EGFL6, EDIL3, PTN, MMP14, VWF, ITGA11, JAM2, COL5A1, IL1B, COL3A1, IGF2, PRKCA, KDR, ITGA1, ADAMTS5, ITGA4, FBLN5, THY1, GPNMB, S1PR3, FAP*
Glycosaminoglycan binding	2.52e−07	35/737	*POSTN, VCAN, THBS1, SLIT3, PTPRS, TNFAIP6, FGF7, FSTL1, BGN, PGF, ADAMTS3, COL23A1, CRISPLD2, COL5A3, RSPO3, PTN, LAYN, LPL, FGFR1, SERPINE2, EFEMP2, LXN, LTBP2, GREM2, COL5A1, PCOLCE, ANXA6, SEMA5A, APOE, ADAMTS5, THBS2, DCN, COL13A1, GPNMB, SULF1*
Platelet‐derived growth factor binding	9.65e−07	8/737	*COL4A1, COL6A1, COL5A1, COL1A2, COL3A1, COL1A1, PDGFRB, PDGFRA*
Growth factor binding	1.3e−06	25/737	*FSTL4, THBS1, IL2RB, NKD2, COL4A1, HTRA3, FLT1, ESM1, FGFR1, IGFBP6, VASN, COL6A1, LTBP2, COL5A1, LRRC32, COL1A2, COL3A1, SRPX2, KDR, A2M, HTRA1, COL1A1, ACVRL1, PDGFRB, PDGFRA*
Heparin binding	2.11e−06	27/737	*POSTN, THBS1, SLIT3, PTPRS, FGF7, FSTL1, PGF, ADAMTS3, COL23A1, CRISPLD2, COL5A3, RSPO3, PTN, LPL, FGFR1, SERPINE2, EFEMP2, LXN, LTBP2, GREM2, COL5A1, PCOLCE, APOE, ADAMTS5, THBS2, COL13A1, GPNMB*
Extracellular matrix binding	3.33e−06	15/737	*TGFBI, THBS1, ITGA7, BGN, ECM1, LGALS1, LRRC15, SSC5D, FBLN2, CD248, SPARCL1, ADAMTS5, DCN, SPARC, NID1*
Metalloendopeptidase activity	4.73e−05	20/737	*MMP7, MMP11, MMP19, ADAM12, ADAMTS3, MMP14, PRSS2, MMP16, ADAM19, MMP9, MMP10, MMP12, ADAMTS10, PAPPA, ADAMTS5, ADAMTS4, MME, MMP1, MMP2, MMP3*

**TABLE 4:** Top 10 most significantly enriched Gene Ontology (GO)‐Cellular Components (CC) pathways in the SSEA‐1^+^ versus SSEA‐1^−^ endometrial epithelial cell populations.

Top 10 enriched GO‐CC pathways	Adjusted *p*	Gene ratio	Enriched genes
Collagen‐containing extracellular matrix	3.12e−26	89/717	*S100A9, POSTN, TGFBI, VCAN, THBS1, SERPING1, LAMB1, AGT, SERPINF1, TGFB1I1, COL4A2, CTHRC1, TIMP2, SERPINH1, COL24A1, COL4A1, BGN, ADAMTS3, EFEMP1, ECM1, COL23A1, LGALS1, COL5A3, EGFL6, COL6A2, EDIL3, F7, COL15A1, WNT2, ANGPTL2, LRRC15, SERPINE2, SSC5D, ANGPTL7, VWF, LAMC3, ADAM19, EFEMP2, MGP, CDH13, MMP9, TIMP3, CTSF, FBLN2, COL6A1, FMOD, TGFB2, LAMA1, CTSC, WNT5B, LTBP2, COL6A3, COL5A1, PCOLCE, ANXA6, A1BG, ADAMTS10, COL8A1, COL1A2, COL3A1, CDH2, APOE, SRPX2, MFAP4, ANGPT2, SPARCL1, AEBP1, ADAMTS5, COL12A1, THBS2, ADAMTS4, DCN, FBLN5, A2M, HTRA1, PLAT, COL13A1, COL7A1, COL1A1, SRPX, GREM1, SULF1, COL5A2, LUM, MMP2, LOXL2, SPARC, NID1, WNT5A*
Endoplasmic reticulum lumen	8.01e−09	48/717	*MSLN, VCAN, THBS1, SERPING1, LAMB1, PRSS23, COL4A2, PDGFC, SERPINH1, WNT4, COL24A1, COL4A1, FSTL1, BACE1, F10, FAM20C, COL23A1, LGALS1, COL5A3, COL6A2, PENK, MXRA8, F7, VGF, COL15A1, RCN3, ARSI, COL6A1, CTSC, WNT5B, COL6A3, FKBP7, COL5A1, FKBP10, COL8A1, COL1A2, COL3A1, CDH2, APOE, SPARCL1, ADAMTS5, COL12A1, TSPAN5, COL13A1, COL7A1, COL1A1, COL5A2, WNT5A*
Collagen trimer	3.48e−08	23/717	*COL4A2, CTHRC1, C1QTNF1, COL24A1, COL4A1, COL23A1, COL5A3, COL6A2, CCBE1, COL15A1, LOX, COL6A1, COL6A3, COL5A1, COL8A1, COL1A2, COL3A1, COL12A1, COL13A1, COL7A1, COL1A1, COL5A2, LUM*
Complex of collagen trimers	8.51e−07	11/717	*COL4A2, COL4A1, COL5A3, COL5A1, COL8A1, COL1A2, COL3A1, COL7A1, COL1A1, COL5A2, LUM*
Fibrillar collagen trimer	0.0001	7/717	*COL5A3, COL5A1, COL1A2, COL3A1, COL1A1, COL5A2, LUM*
Basement membrane	0.0002	18/717	*TGFBI, LAMB1, SERPINF1, COL4A2, COL4A1, EGFL6, COL15A1, LAMC3, EFEMP2, TIMP3, LAMA1, COL5A1, COL8A1, THBS2, COL7A1, LOXL2, SPARC, NID1*
Platelet alpha granule lumen	0.003	13/717	*THBS1, SERPING1, PROS1, HGF, SRGN, VWF, TGFB2, A1BG, VEGFC, IGF2, ISLR, A2M, SPARC*
Platelet alpha granule	0.007	15/717	*THBS1, SERPING1, PROS1, HGF, SRGN, SERPINE2, VWF, TGFB2, A1BG, VEGFC, IGF2, ISLR, THBS2, A2M, SPARC*
Golgi lumen	0.008	16/717	*HS3ST1, VCAN, MMP11, PROS1, WNT4, BGN, GOLIM4, F10, F7, MMP14, MMP16, FMOD, WNT5B, DCN, LUM, WNT5A*
External side of plasma membrane	0.013	37/7171	*TNF, IGLL5, PDCD1LG2, THBS1, ITGA7, ASGR1, IL2RB, CD274, FCER2, CCR1, CCR7, GFRA2, ITGA10, F10, CCR10, ITGA8, ABCA1, CHRNA4, LAG3, WNT2, SERPINE2, CLEC2B, ITGA11, ANPEP, CDH13, CNTFR, HEG1, CD248, IL7R, KDR, ITGA1, ITGA4, ANTXR1, THY1, CTSK, IL13RA2, PDGFRA*

#### 3.2.2 Hallmark and KEGG Enrichment Pathways

**FIGURE 3:**
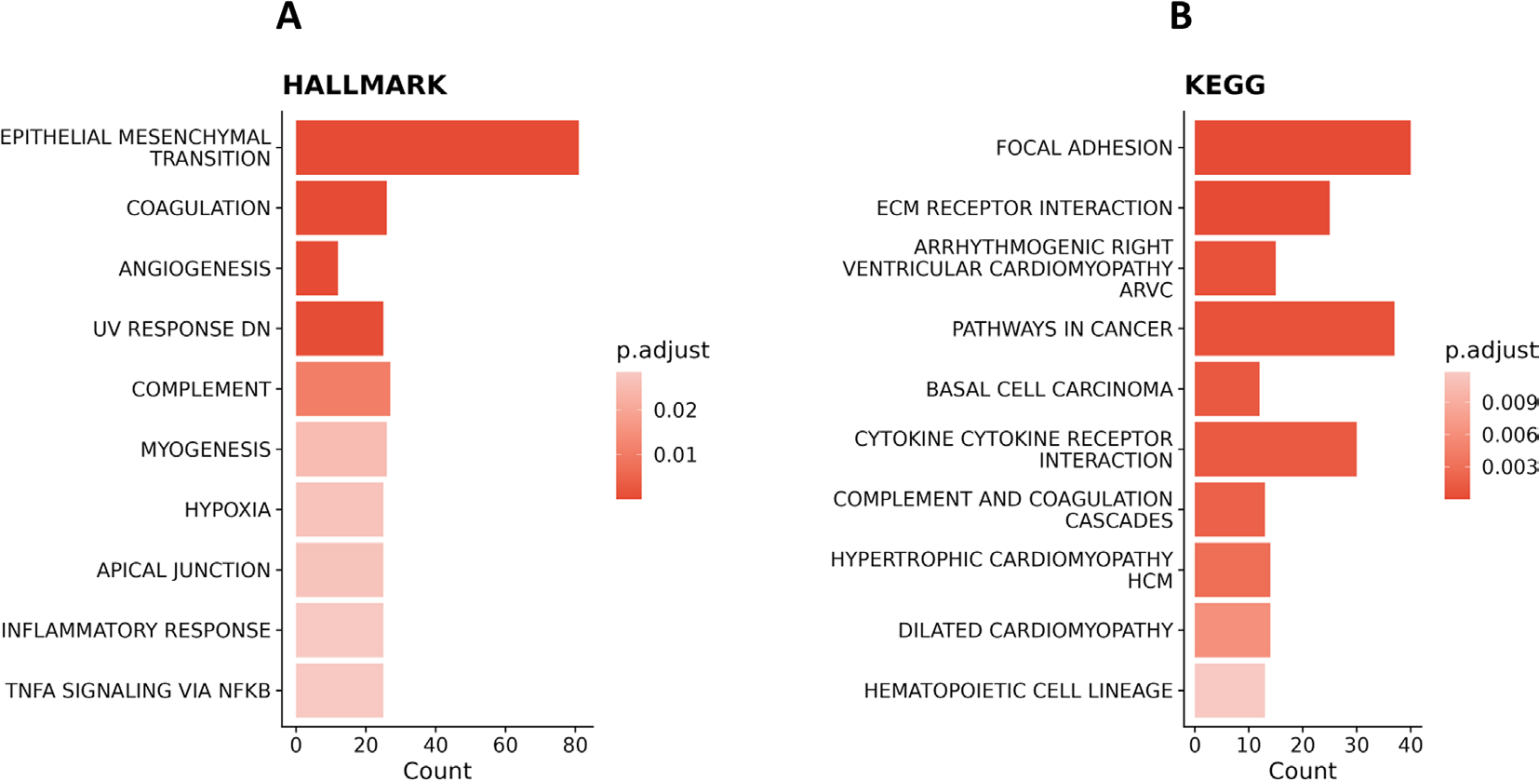
Gene enrichment analysis (A) Bar chart of top 10 enriched Hallmark pathways (B) Bar chart of top 10 enriched KEGG pathways.

Hallmark and KEGG pathways demonstrated that SSEA‐1^+^ EECs are important in pathways related to epithelial‐to‐mesenchymal transition and angiogenesis. The top three most significantly enriched Hallmark pathways relevant to regeneration and remodeling included “epithelial mesenchymal transition” (81 genes, FDR = 2.8e−41), “coagulation” (26 genes, FDR = 0.0001), and “angiogenesis” (12 genes, FDR = 0.0001) (Figure 3A and Table 5). The top three most significantly enriched KEGG pathways included “focal adhesion” (40 genes, FDR = 3.16e‐11), “ECM receptor interaction” (25 genes, FDR = 8.94e−11), and “arrhythmogenic right ventricular cardiomyopathy ARVC” (15 genes, FDR = 0.00047) (Figure 3B and Table 6).

**TABLE 5:** Top 10 most significantly enriched Hallmark pathways in the SSEA‐1^+^ versus SSEA‐1^−^ endometrial epithelial cell populations.

Top 10 enriched hallmark pathways	FDR	Gene ratio	Enriched genes
Epithelial mesenchymal transition	2.8e−41	81/317	*AREG, POSTN, TGFBI, VCAN, DKK1, THBS1, SLIT3, COL4A2, CTHRC1, GJA1, SGCB, ADAM12, SERPINH1, WIPF1, COL4A1, FSTL1, BGN, OXTR, SFRP4, ECM1, MYLK, LGALS1, PDLIM4, COL5A3, MYL9, ACTA2, COL6A2, EDIL3, GLIPR1, GAS1, NTM, MMP14, GEM, LRRC15, SERPINE2, PRSS2, EFEMP2, ANPEP, MGP, TIMP3, FBLN2, LOX, FMOD, LAMA1, COL6A3, TNFRSF11B, COL5A1, PCOLCE, SNAI2, VEGFC, COL1A2, COL3A1, CDH2, RGS4, SPOCK1, PMP22, COL12A1, TPM2, THBS2, DAB2, DCN, FBLN5, THY1, HTRA1, COL7A1, CDH11, COL1A1, PRRX1, VIM, GREM1, MMP1, COL5A2, LUM, MMP2, FAP, LOXL2, INHBA, SPARC, PDGFRB, WNT5A, MMP3*
Coagulation	0.0001	26/317	*CTSE, MMP7, THBS1, MMP11, SERPING1, PROS1, PRSS23, OLR1, F10, DUSP6, MMP14, VWF, MMP9, TIMP3, MMP10, C1S, C1R, A2M, HTRA1, PLAT, CTSK, MMP1, F2RL2, MMP2, SPARC, MMP3*
Angiogenesis	0.0001	12/317	*POSTN, VCAN, OLR1, FSTL1, LPL, FGFR1, STC1, KCNJ8, CCND2, COL3A1, COL5A2, LUM*
UV response dn	0.0003	25/317	*FHL2, MAGI2, CDK13, CITED2, GJA1, GRK5, FZD2, RND3, EFEMP1, TFPI, HAS2, MMP16, SNAI2, COL1A2, COL3A1, RGS4, AKT3, PMP22, PRKCA, DAB2, PRKAR2B, FBLN5, COL1A1, COL5A2, PDGFRB*
Complement	0.0106	27/317	*S100A9, GZMA, SERPING1, KCNIP2, GNB4, ZFPM2, COL4A2, CASP1, TIMP2, OLR1, CEBPB, F10, SH2B3, KLK1, PRSS3, DUSP6, F7, MMP14, KCNIP3, CDH13, CTSC, MMP12, C1S, C1R, DOCK4, PLAT, ZEB1*
Myogenesis	0.0253	26/317	*ITGA7, KCNH1, COL4A2, ADAM12, PDE4DIP, SGCA, MYLK, COL6A2, PFKM, MYH11, COL15A1, PPP1R3C, MRAS, CDH13, HSPB2, CACNA1H, COL6A3, EFS, COL3A1, MYBPH, AEBP1, TPM2, COX7A1, BDKRB2, COL1A1, SPARC*
Hypoxia	0.2701	25/317	*HS3ST1, TGFBI, DPYSL4, SLC2A3, CITED2, EXT1, SAP30, NFIL3, CSRP2, BGN, PPARGC1A, MT2A, PGF, AMPD3, STC1, PPP1R3C, LXN, LOX, COL5A1, CHST2, ADM, PRKCA, DCN, TMEM45A, SRPX*
Apical junction	0.2715	25/317	*CLDN8, DSC1, TGFBI, VCAN, CD274, NRXN2, TRO, SGCE, CLDN5, ITGA10, NEXN, MYL9, LAYN, CDH4, CALB2, ACTG2, VWF, MMP9, TNFRSF11B, FSCN1, AKT3, ADAMTS5, THY1, CDH11, MMP2*
Inflammatory response	0.2839	25/317	*IL18, CALCRL, KCNMB2, IL2RB, OLR1, TNFSF15, TNFAIP6, PTPRE, CCR7, AXL, ABCA1, SLC1A2, MMP14, HAS2, NAMPT, CSF3, BDKRB1, CHST2, IL1B, PTGIR, ADM, PDPN, IL7R, PDE4B, INHBA*
TNF‐a signaling via NKF‐b	0.2839	25/317	*AREG, IL18, TNF, ETS2, SLC2A3, OLR1, NFIL3, MARCKS, TNFAIP6, EGR2, PTPRE, CEBPB, NINJ1, KLF9, ABCA1, GEM, FJX1, NAMPT, SLC16A6, GFPT2, BMP2, IL1B, IL7R, PDE4B, INHBA*

**TABLE 6:** Top 10 most significantly enriched KEGG pathways in the SSEA‐1^+^ versus SSEA‐1^−^ endometrial epithelial cell populations.

Top 10 enriched KEGG pathways	FDR	Gene ratio	Enriched genes
Focal adhesion	3.16e−11	40/276	*THBS1, ITGA7, LAMB1, MAPK8, COL4A2, PDGFC, SHC4, COL4A1, HGF, FLT1, PGF, ITGA10, ITGA8, MYLK, COL5A3, MYL9, COL6A2, VWF, LAMC3, VAV3, ITGA11, CCND2, SHC3, COL6A1, LAMA1, COL6A3, COL5A1, VEGFC, COL1A2, COL3A1, AKT3, PRKCA, KDR, ITGA1, THBS2, ITGA4, COL1A1, COL5A2, PDGFRB, PDGFRA*
ECM receptor interaction	8.94e−11	25/276	*CD47, THBS1, ITGA7, LAMB1, COL4A2, COL4A1, GP1BB, ITGA10, ITGA8, COL5A3, COL6A2, VWF, LAMC3, ITGA11, COL6A1, LAMA1, COL6A3, COL5A1, COL1A2, COL3A1, ITGA1, THBS2, ITGA4, COL1A1, COL5A2*
Arrhythmogenic right ventricular cardiomyopathy arvc	0.00047	15/276	*ITGA7, GJA1, SGCB, SGCA, CACNA2D4, TCF7, ITGA10, ITGA8, CACNA1F, ITGA11, CACNA2D1, CDH2, LEF1, ITGA1, ITGA4*
Pathways in cancer	0.00048	37/276	*FGF18, KITLG, LAMB1, MAPK8, COL4A2, CSF1R, WNT4, COL4A1, HGF, FGF7, TCF7, FZD2, PGF, GLI1, GLI3, SMO, RARB, FGFR1, WNT2, LAMC3, MMP9, TGFB2, LAMA1, FZD4, WNT5B, BMP2, VEGFC, LEF1, AKT3, PRKCA, AR, RUNX1T1, MMP1, MMP2, PDGFRB, PDGFRA, WNT5A*
Basal cell carcinoma	0.00101	12/276	*WNT4, TCF7, FZD2, GLI1, GLI3, SMO, WNT2, FZD4, WNT5B, BMP2, LEF1, WNT5A*
Cytokine receptor interaction	0.00112	30/276	*IL18, TNF, KITLG, TNFRSF8, IL2RB, PDGFC, CSF1R, TNFSF15, HGF, FLT1, CCR1, CCR7, CCR10, TNFRSF6B, CCL26, CSF3, IL24, CNTFR, TGFB2, TNFRSF11B, BMP2, VEGFC, IL1B, IL7R, KDR, IL11, ACVRL1, INHBA, PDGFRB, PDGFRA*
Complement and coagulation cascades	0.00176	13/276	*SERPING1, PROS1, F10, TFPI, F7, VWF, BDKRB1, F2R, C1S, C1R, BDKRB2, A2M, PLAT*
Hypertrophic cardiomyopathy hcm	0.00289	14/276	*TNF, ITGA7, SGCB, SGCA, CACNA2D4, ITGA10, ITGA8, CACNA1F, ITGA11, CACNA2D1, TGFB2, ITGA1, TPM2, ITGA4*
Dilated cardiomyopathy	0.00616	14/276	*TNF, ITGA7, SGCB, SGCA, CACNA2D4, ITGA10, ITGA8, CACNA1F, ITGA11, CACNA2D1, TGFB2, ITGA1, TPM2, ITGA4*
Hematopoietic cell lineage	0.01178	13/276	*TNF, KITLG, CSF1R, FCER2, GP1BB, CSF3, ANPEP, IL1B, IL7R, ITGA1, ITGA4, MME, IL11*

### 3.3 Ingenuity Pathway Analysis suggests that SSEA‐1^+^ EECs have a Distinct Phenotype and Function

#### 3.3.1 IPA Diseases and Bio Functions

Using IPA we were further able to biologically interpret the DEGs using the extensive knowledge base contained within the platform. Firstly, IPA generated associations between the DEGs and specific “diseases and disorders”, “molecular and cellular functions”, and “physiological system development and function”. The top five within each of these categories are highlighted in Table 7. These suggest that SSEA‐1^+^ EECs are important for tissue homeostasis including functions such as tissue proliferation, development, maintenance, survival, and angiogenesis.

**TABLE 7:** Top diseases and biofunctions in the SSEA‐1^+^ versus SSEA‐1^−^ endometrial epithelial cell populations.

Top diseases and biofunctions		
Diseases and disorders	*p* range	Number of molecules
Cancer	6.02e−07–4.69e−32	937
Organismal injury and abnormalities	6.02e−07–4.69e−32	945
Reproductive system disease	4.59e−07–4.00e−31	789
Cardiovascular disease	5.54e−07—6.48e−29	406
Skeletal and muscular disorders	5.79e−07–6.22e−28	415

Molecular and cellular functions	*p* range	Number of molecules
Cellular movement	6.06e−07–1.13e−52	442
Cellular development	4.76e−07–1.34e−27	534
Cellular function and maintenance	2.51e−07–2.21e−23	419
Cellular growth and proliferation	4.76e−07–2.21e−23	497
Cell‐to‐cell signaling and interaction	6.06e−07–9.38e−22	384

Physiological system development and function	*p* range	Number of molecules
Cardiovascular system development and function	5.97e−07–7.44e−59	354
Organismal development	5.79e−07–4.04e−56	579
Embryonic development	5.45e−07–1.68e−34	431
Organismal survival	8.63e−14–4.85e−32	416
Connective tissue development and function	5.22e−07–2.09e−30	310

Further exploration of “diseases and functions” associated with the DEGs, predicted “organismal death” (*z*‐score = 14.60, FDR = 4.85e−32) and “dysgenesis” (*z*‐score = 7.42, FDR = 8.41e−13) to be within the top activated biofunctions with a *z*‐score > 2 and FDR < 0.05. The bottom *z*‐scores (with *z*‐scores < −2 and FDR < 0.05) predicted inhibition of “size of body” (*z*‐score = −9.28, FDR = 9.09e−15), “cell movement” (*z*‐score = −8.17, FDR = 1.13e−52), “cell movement of tumor cell lines” (*z*‐score = −7.97, FDR = 1.15e−32), “migration of cells” (*z*‐score = −7.81, FDR = 2.54e−49) and “migration of tumor cell lines” (*z*‐score = −7.67, FDR = 1.44e−28). The IPA downstream effects analysis table (Table S6) shows the tabular output that predicts “organismal death” as the predicted top activated biofunctions, based on the observed changes in gene expression within the SSEA‐1^+^ EECs.

#### 3.3.2 IPA Canonical Pathway Analysis

Using the QIAGEN IPA knowledge base, well‐characterized metabolic and cell signaling pathways were explored based on overlap from the DEGs. IPA's canonical pathway analysis suggests SSEA‐1^+^ EECs to have a tumor suppressor and homeostatic function and a reduced hormone responsive phenotype.

Significant overlap was found between the genes involved within five canonical pathways which were predicted to be activated within the SSEA‐1^+^ EECs. These included “RhoGDI signaling” (*p*‐value = 6.52e−04, *z*‐score = 3.21), “endocannabinoid cancer inhibition pathway” (*p*‐value = 6.79e−03, *z*‐score = 3.21), “PTEN signaling” (*p*‐value = 1.81e−02, *z*‐score = 1.74), “chaperone mediated autophagy signalling pathway” (*p*‐value = 0.04, *z*‐score = 2.83), and “CDX gastrointestinal cancer signalling pathway” (*p*‐value = 8.69e−05, *z*‐score 2.29). An example of the “PTEN signalling pathway” and “endocannabinoid cancer inhibition pathway” are shown in Figure S1A,B, highlighting the tumor suppressor pathways that are predicted to be activated within the SSEA‐1^+^ EECs.

Overall, an overwhelming majority of 83 significantly overlapping canonical pathways were predicted to be inhibited in the SSEA‐1^+^ EECs. The top five predicted to be most inhibited within the SSEA‐1^+^ EECs were “molecular mechanisms of cancer” (*p*‐value = 5.55e−06, *z*‐score = −7.54), “FAK signaling” (*p*‐value = 1.44e−08, z‐score = −7.39), “pulmonary fibrosis signalling pathway” (*p*‐value = 8.15e−22, *z*‐score = −7.18), “phagosome formation” (*p*‐value = 6.77e−04, *z*‐score = −6.93) and “CREB signalling in neurons” (*p*‐value = 2.5e−04, *z*‐score = −6.87).

The canonical “estrogen receptor signalling” pathway is also seen to significantly overlap with the DEGs (*p*‐value = 4.79e−03); however, using the IPA Knowledge Base, this pathway is predicted to be inhibited within the SSEA‐1^+^ EEC population (z‐score = −4.16). This pathway is seen to control functions such as “tumor cell proliferation”, “tumor EMT”, “metastasis”, “cell proliferation” and “migration of tumour cells”, which are all predicted to be inhibited due to the downregulation of DEGs, mainly matrix metallopeptidases (MMPs) involved in this pathway. The pathway “estrogen receptor signalling” and genes involved are detailed in Figure S2.

## 4 SSEA‐1^+^ EECs Tumor Suppressor Activity May Be Controlled by the Upregulation of SAM Pointed Domain Containing ETS Transcription Factor (SPDEF), Inhibition of Transforming Growth Factor‐Beta 1 (TGFB1) and Some Known Regulators of Endometrial Carcinogenesis

“Upstream analysis” within IPA was used to predict significantly activated and inhibited upstream regulators within the SSEA‐1^+^ EEC population and their associated mechanistic and causal networks, which may explain the observed expression changes. Upstream regulator analysis allows determination of likely upstream regulators that are connected to the DEGs either through direct or indirect relationships. To understand the relationship between upstream regulators and DEGs within the dataset, mechanistic and causal networks were used. As upstream regulators are not necessarily independent of one another, mechanistic networks allowed us to hypothesize other connecting regulators that are likely to be part of the same signaling or causal mechanism, whereas causal networks allowed the detection of novel master upstream regulators [21].

**FIGURE 4:**
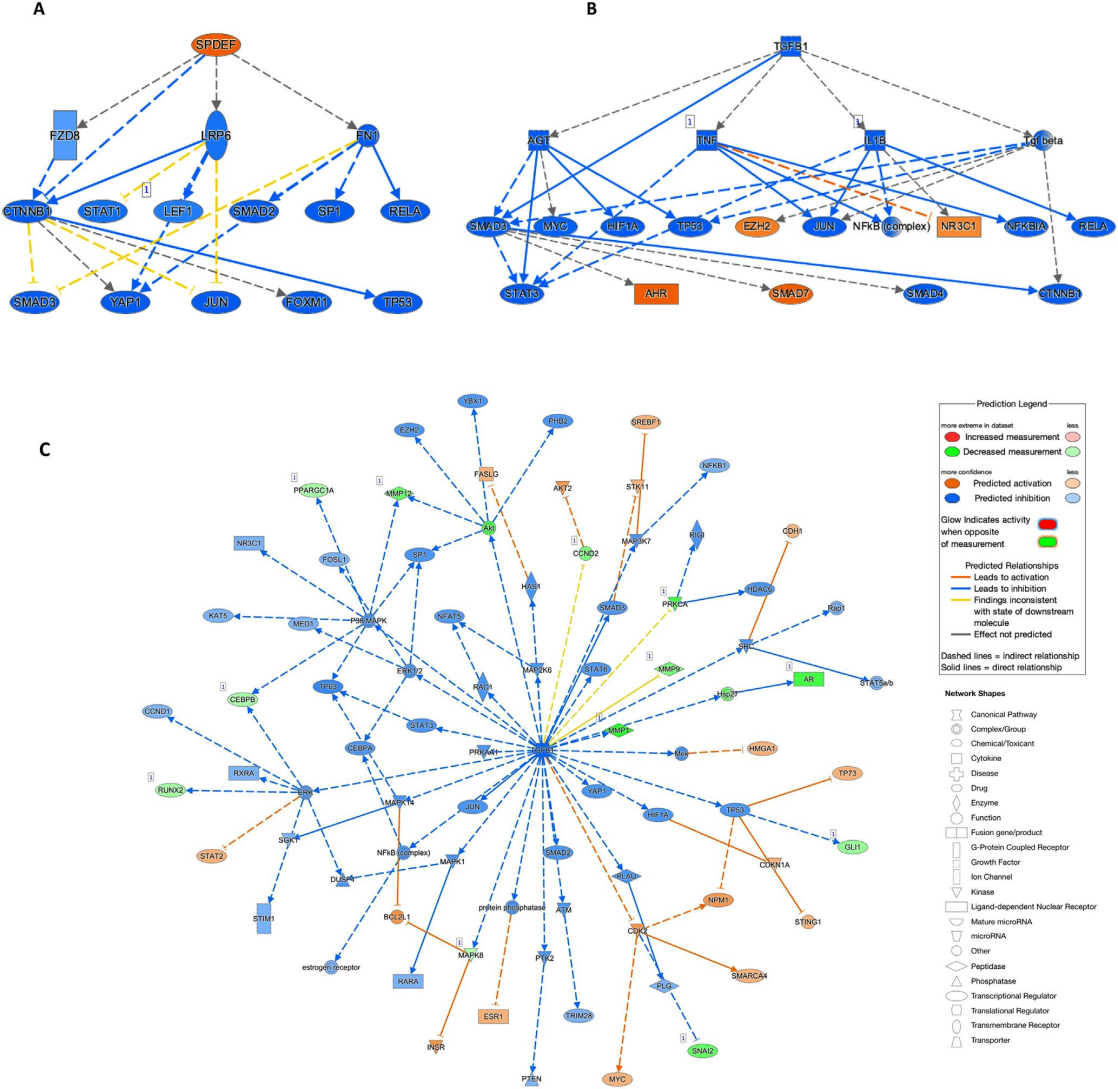
IPA's upstream regulator analysis. Mechanistic networks for (A) SPDEF and (B) TGFB1 displaying other predicted connecting upstream regulators likely to be part of the same signaling mechanism. (C) Causal network for TGFB1 predicted to be a master regulator and its downstream targets. Orange nodes represents predicted activation. Green nodes represent significantly downregulated DEGs within the dataset. Solid lines represent a direct relationship and dashed lines represent indirect relationships between nodes. Orange lines predict a relationship resulting in activation and blue lines predict a relationship resulting in inhibition.

Within our dataset, the transcription factor SPDEF (*p*‐value of overlap = 1.05e−10, *z*‐score = 4.30) was predicted to be one of the top‐most activated upstream regulators, with significant overlap of their target molecules seen within this microarray DEGs dataset. On the contrary, TGFB1 is predicted to be one of the top‐most inhibited upstream regulators with the most significant number of target molecules of overlap within our dataset (*p*‐value of overlap = 8.64e−40, *z*‐score = −8.20). The mechanistic networks of SPDEF and TGFB1 and their downstream target molecules within our DEGs are displayed within Figure 4A,B. When exploring causal networks for a master upstream regulator, TGFB1 was also found to be predicted as the top‐most inhibited master regulator for the DEGs (*p*‐value of overlap = 8.64e−40, *z*‐score = −8.20) (Figure 4C). In addition, upstream regulator analysis revealed overlap between several upstream regulators predicted to control the biological mechanisms involved in SSEA‐1^+^ EECs and regulators known to be involved in endometrial carcinogenesis [26, 27, 28]. These regulators include *ARID2, KRAS, CTNNB1, PIK3CA*, and *TP63*.

### 4.1 DEGs Identified via Microarray Analysis are Confirmed with RT‐qPCR and their Gene Products are Co‐Localized with SSEA‐1: Internal Validation

**FIGURE 5:**
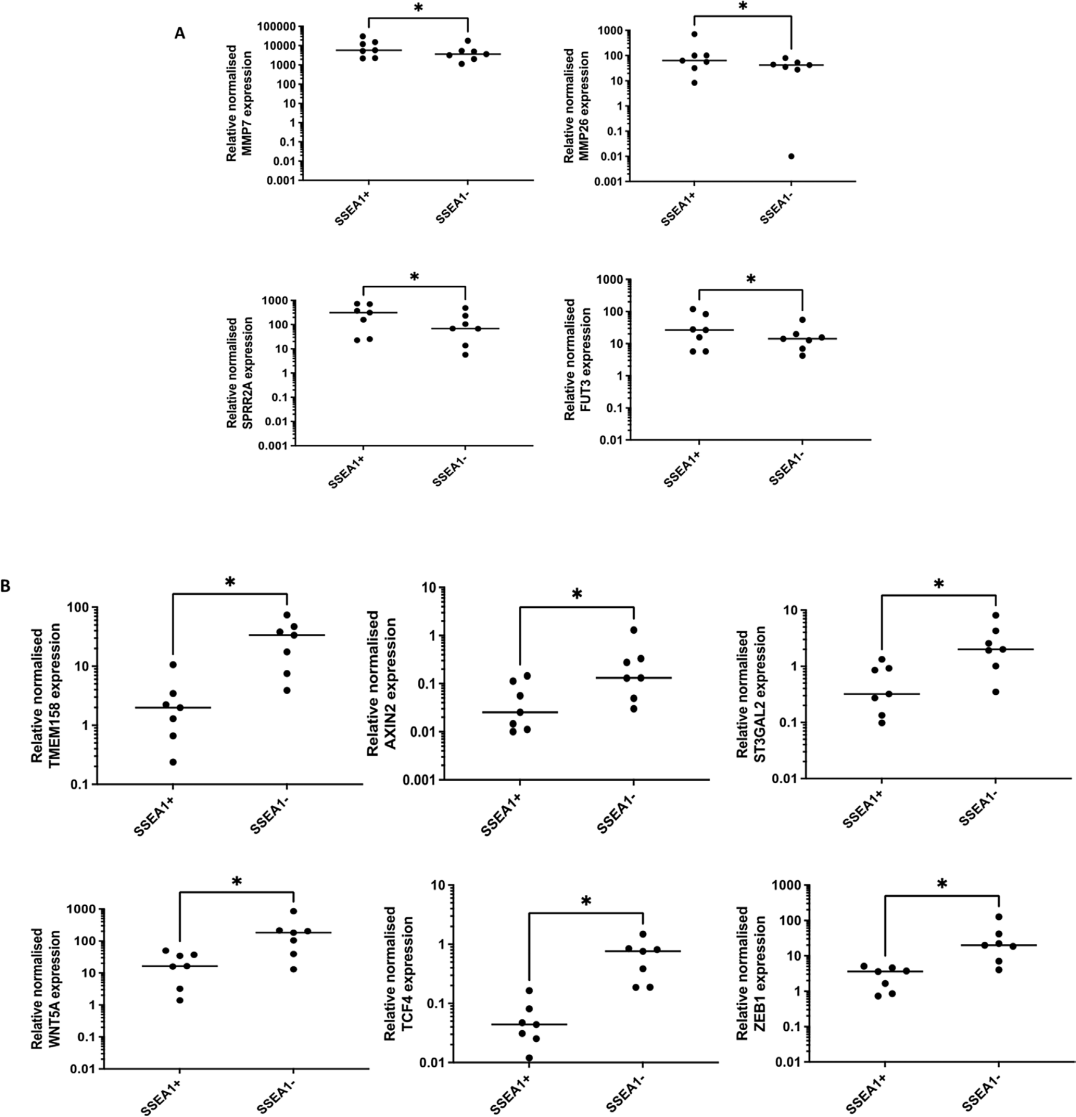
Quantitative real‐time PCR analysis of transcripts validating microarray gene expression data for (A) upregulated genes: *MMP7*, *MMP26*, *SPRR2A*, *FUT3* and (B) downregulated genes: *TMEM158*, *AXIN2*, *ST3GAL2*, *WNT5A*, *TCF4*, *ZEB1*. Relative normalized mRNA expression was confirmed to be significantly greater in SSEA‐1^+^ EECs for *MMP7* (*p* = 0.016), *MMP26* (*p* = 0.016), *SPRR2A* (*p* = 0.016) and *FUT3* (*p* = 0.031). Relative normalized mRNA expression was confirmed to be significantly lower in SSEA‐1^+^ EECs for *TMEM158* (*p* = 0.016), *AXIN2* (*p* = 0.016), *ST3GAL2* (*p* = 0.016), *WNT5A* (*p* = 0.016), *TCF4* (*p* = 0.016) and *ZEB1* (*p* = 0.016). significant differences *p* < 0.05 are depicted with “*”.

**FIGURE 6:**
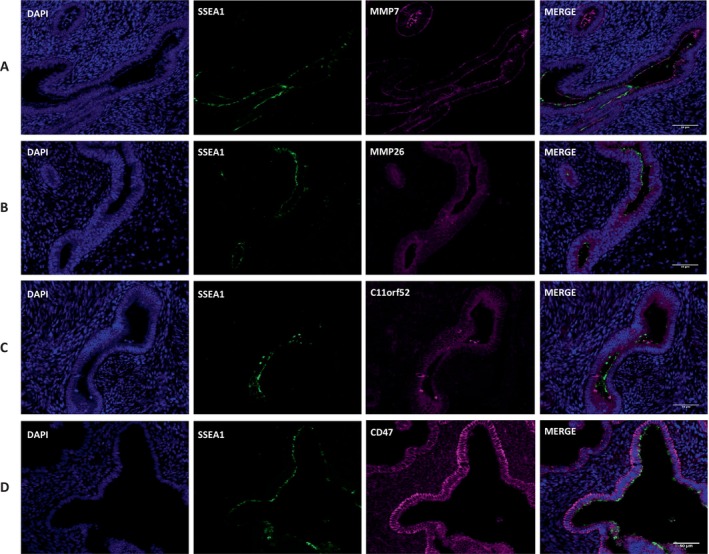
Representative micrographs show co‐localization of (A) *MMP7*, (B) *MMP26*, (C) *C11orf52* and (D) CD47 with SSEA‐1 within the basal glands of the endometrium using dual immunofluorescence. Scale bars = 50 μm.

Microarray data was validated using RT‐qPCR to assess the mRNA expression levels of a selection of significantly up‐and downregulated genes. *MMP7* (*p* = 0.016), *MMP26* (*p* = 0.016), *SPRR2A* (*p* = 0.016) and *FUT3* (*p* = 0.03) showed significantly higher mRNA expression levels within the SSEA‐1^+^ EECs compared with the SSEA‐1^−^ EECs (Figure 5A). In contrast, significantly lower gene expression levels for *TMEM158*, *AXIN2*, *ST3GAL2*, *WNT5A*, *TCF4*, and *ZEB1* were confirmed within the SSEA‐1^+^ EECs compared to the SSEA‐1^−^ EECs (*p* = 0.016; Figure 5B). Dual‐IF staining of human endometrial tissue sections confirmed coexpression of SSEA‐1 with proteins encoded by significantly upregulated genes identified by microarray analysis, including MMP7, MMP26, C11orf52, and CD47 (Figure 6).

### 4.2 Upregulated DEGs MMP7 and MMP26 are Predominantly Expressed by Glandular SOX9 ^+^ and SOX9 ^+^/LGR5 ^+^ EECs within Single‐Cell Transcriptomic Data: External Validation

**FIGURE 7:**
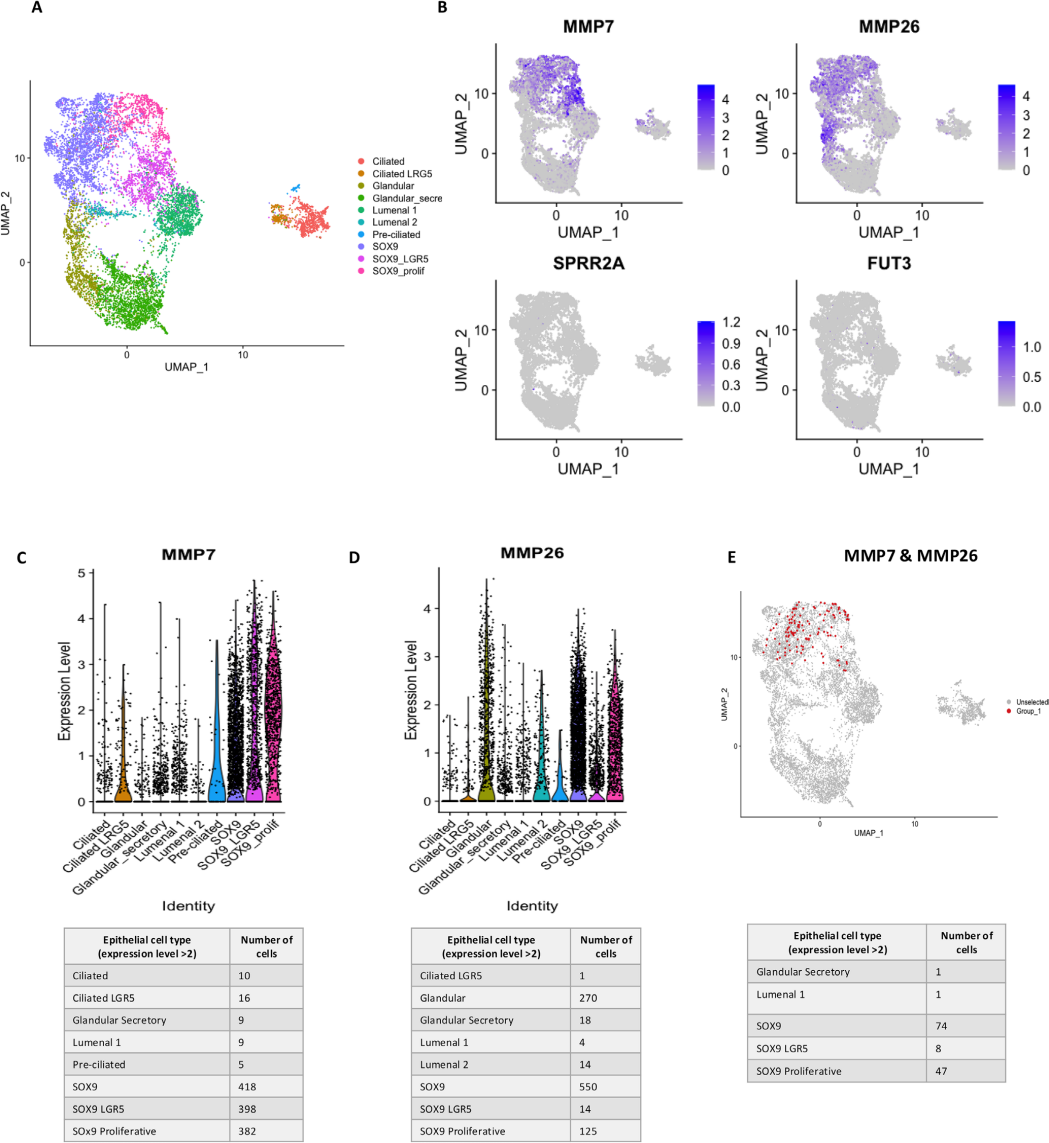
UMAP of single‐cell EEC transcriptomic data published by Garcia et al. [22]. (A) UMAP of all subclustered and subsampled epithelial populations. (B) UMAP mapping *MMP7*, *MMP26*, *SPRR2A* and *FUT3* to single‐cell EEC clusters. (C) Violin plots of *MMP7* and (D) *MMP26* expressing single‐cell EECs and number of epithelial cell types expressing each marker at an expression level > 2. (E) UMAP of cells co‐expressing *MMP7* and *MMP26* at an expression level > 2, mapped to single‐cell EEC clusters.

DEGs *MMP7, MMP26, SPRR2A*, and *FUT3* were found to be significantly upregulated within SSEA‐1^+^ EECs by both microarray gene expression analysis and RT‐qPCR. They were mapped to the publicly available single‐cell transcriptomic dataset published by Garcia et al. [22] as a further method of external validation to assess the epithelial cell types that express these genes (Figure 7A,B). At an expression level > 2, *MMP7* is predominantly expressed by *SOX9* ^+^ (*SOX9* ^+^ EECs: 418 cells; *SOX9* ^+^ proliferative EECs: 382 cells) and *SOX9* ^+^/*LGR5* ^+^ EECs mainly within the proliferative phase (*SOX9* ^+^/*LRG5* ^+^ EECs: 398 cells) and within a much smaller number of ciliated *LGR5* ^+^ EECs (ciliated EECs: 10 cells; ciliated *LGR5* ^+^ EECs: 16 cells) (Figure 7C). At an expression level > 2, *MMP26* was found to be predominantly expressed by glandular *SOX9* ^+^ EECs within the proliferative phase (glandular EECs: 270 cells; *SOX9* ^+^ EECs: 550 cells; *SOX9* ^+^ proliferative EECs: 125 cells) (Figure 7D). When combined, *SOX9* ^+^ cells within the proliferative phase were found to predominantly express both *MMP7* and *MMP26* at an expression level > 2 (*SOX9* ^+^ EECs: 74 cells; *SOX9* ^+^ proliferative EECs: 47 cells; *SOX9* ^+^ */LRG5* ^+^ EECs: 8 cells), suggesting co‐expression of SSEA‐1^+^ and SOX9^+^ EECs, in keeping with previous literature [10] (Figure 7E).

### 4.3 Endometrial Epithelial Organoids Exposed to Ovarian Hormones show Distinct Phenotypic Protein Expression Patterns for SSEA‐1

**FIGURE 8:**
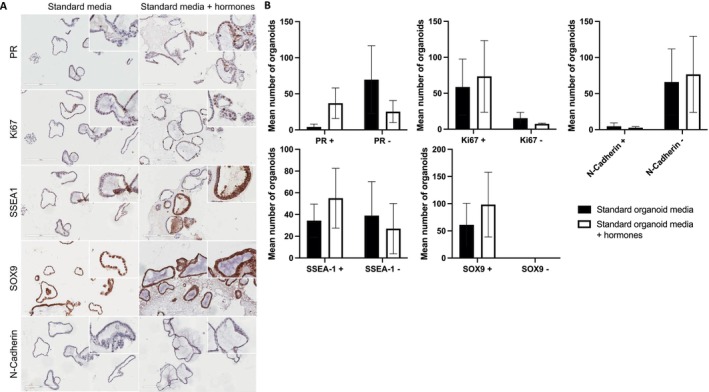
Immunohistochemical staining for PR, Ki‐67, SSEA‐1, SOX9 and N‐Cadherin in endometrial epithelial organoids exposed to either standard media or standard media plus hormones. (A) Representative micrographs of immunohistochemical (IHC) staining for each protein. Scale bar is 200 μm. (B) IHC scoring displayed as the mean number of positively or negatively stainedg endometrial epithelial organoids, Error bars represent the standard error of the mean (SEM).

The ovarian hormone milieu in the secretory phase of the menstrual cycle induces endometrial cell differentiation [29]. To simulate this in vitro, human endometrial epithelial organoids were exposed to hormonal stimuli. The expression of proposed EEC SPC markers SSEA‐1, SOX9 and N‐cadherin, PR as a marker of estrogen response, and the proliferation marker Ki‐67 were assessed (Figure 8A,B). Compared with controls (standard media), hormone treatment resulted in a higher mean number of organoids expressing SSEA‐1 (34.3 out of 73.3 (46.8%) versus 55 out of 82 (67.1%)), PR (4 out of 73.7 (5.4%) versus 37 out of 62.3 (59.4%)), and Ki‐67 (58.7 out of 74 (79.3%) versus 73.3 out of 80.7 (90.8%)), while a lower proportion expressed N‐cadherin (4.7 out of 70.7 (6.6%) versus 2.7 out of 79.3 (3.4%)). SOX9 was ubiquitously present across all organoids exposed to both culture conditions (69 out of 69 (100%) versus 98.33 out of 98.33 (100%)).

## 5 Discussion

This study reports the transcriptional profile of the first postulated human endometrial epithelial SPC population, SSEA‐1^+^ EECs, which agrees with their expected SPC function. Although it has been hypothesized that the basalis EECs have a role in endometrial regeneration, our data show for the first time that the transcriptional signature of SSEA‐1^+^ EECs is in keeping with the expected SPC phenotypic and functional pathways. Relative to other EECs, SSEA‐1^+^ EECs possess a distinct transcriptional signature that highlights their importance in regeneration, remodeling, and vascularization of the human endometrium. We have employed multiple approaches in pathway and gene set analysis (GO, KEGG, Hallmark and IPA canonical pathways) which cover a wide range of overlapping comprehensive data repositories and produce robust, corroborative results suggesting that the SSEA‐1^+^ EEC transcriptional profile is consistent with SPC function. These findings are pivotal to guide future research to understand and target the specific pathways for diagnosis and therapy for gynecological conditions. For example, the SPC function, responsible for endometrial regeneration, is postulated to be perturbed in persistent endometrial pathologies; thus, examinations of the SSEA‐1^+^ EEC transcriptome in diseased endometrium will provide clinically impactful results.

Pathway analysis revealed important processes related to “vascular and mesenchymal development”, “extracellular matrix structural constituent”, “angiogenesis”, “TNFα and Notch signaling” related to SSEA‐1^+^ EECs. These were associated with biological functions, such as apoptosis, the inhibition of cell movement, cell proliferation and cell growth, supportive of the quiescent SPC phenotype postulated of the SSEA‐1^+^ EECs. Corresponding with the findings of Valentijn et al. [2], significantly reduced *PR* (a well‐established downstream target of estrogen action) expression levels and a downregulation of estrogen receptor pathway were also confirmed.

Abnormal endometrial SPC populations have been implicated in gynecological pathologies such as endometriosis, endometrial cancer, and recurrent reproductive failure [8, 10, 30]. Understanding the biological processes and functional pathways specific to SSEA‐1^+^ EECs in the normal endometrium is essential for improving our understanding of endometrial physiology as well as in the diseased state. In this respect, the identification of important biofunctions in SSEA‐1^+^ EECs, such as the inhibition of tumor cell invasion and migration, in addition to maintaining endometrial homeostasis, is notable. The activation of functional pathways such as “PTEN signaling” and “endocannabinoid cancer inhibition” in IPA's “diseases and functions” and “canonical pathways” analysis demonstrates the fine equilibrium that is expected by a progenitor cell population. These would support rapid and robust proliferation at the time of regeneration, while providing protection from hyperplastic or carcinogenic transformation [31, 32, 33, 34, 35]. Inhibition of the “estrogen receptor signalling pathway” within SSEA‐1^+^ EECs also confirms their restrained hormone responsiveness, a feature that is important to prevent abnormal endometrial growth and carcinogenesis [36].

Our microarray data was validated using multiple methods. Ten significantly up‐or downregulated genes from the microarray analysis were internally validated using RT‐qPCR on the same sample set, demonstrating concordance. Additionally, co‐localization of the gene products of four selected genes with SSEA‐1^+^ EECs was demonstrated in an external biological cohort of endometrial samples. A further external *in silico* validation was conducted using a publicly available single‐cell transcriptomics dataset, which maps the epithelial subtypes within the human endometrium [22]. The significantly upregulated DEGs (*MMP7, MMP26, and SPRR2A*) of SSEA‐1^+^ EECs that we had already validated with RT‐qPCR were mapped *in silico* with *FUT3*. Since there is no direct gene coding for the glycoprotein SSEA‐1, *FUT3* may be an important surrogate marker for SSEA‐1^+^ EECs [37, 38]. While *SPRR2A* and *FUT3* expression were sparse and at low levels within the Garcia‐Alonso et al. dataset [22], *MMP7* and *MMP26* showed much stronger expression mainly localized to *SOX9* ^+^ and *SOX9* ^+^ */LGR5* ^+^ EECs. This also corresponds to the previously described progenitor cell phenotype of SSEA‐1^+^/SOX9^+^ co‐localization within basal glandular epithelial cells by Valentijn et al. [2]. *MMP7, MMP26, and SPRR2A* are seen to be important for wound and mucosal repair, and therefore may contribute to epithelial barrier restoration following menstruation [39, 40].

In 2012, Nguyen et al. used a gene microarray approach to assess the transcriptome of highly purified EpCAM^+^ cells from post‐menopausal endometrium (proposed to represent the pre‐menopausal basalis only) versus epithelial cells from pre‐menopausal full‐thickness endometrium (which contained functionalis and basalis cells). Our studies are not comparable methodologically, and thus, expectedly, there was no overall correlation found between the two datasets (spearman correlation coefficient = −0.04). However, we did find an overlap between seven upregulated and 33 downregulated DEGs across the two microarray datasets (Figure S3). Of the genes validated by RT‐qPCR, the significantly upregulated genes *MMP7* and *FUT3* and significantly downregulated genes *TMEM158* and *ZEB1* correlated with the post‐menopausal samples included by Nguyen et al. [12]. Interestingly, while Nguyen et al. found nuclear *AXIN2* to be upregulated in post‐menopausal endometrium compared to pre‐menopausal endometrium, we found *AXIN2*, another proposed endometrial progenitor marker, to be downregulated in the pre‐menopausal SSEA‐1^+^ EECs. Further exploration of EECs of pre‐and postmenopausal endometrium is required in the future to complete our understanding of the discerning features relevant to SPC populations exposed to these two different hormonal milieus.

The effect of hormones in influencing SSEA‐1^+^ EECs were examined using an endometrial epithelial organoid model. Exposure to a combination of oestradiol and progestin demonstrated stronger immunostaining of PR and Ki‐67, thus, estrogen‐responsive proliferation. Although the inhibited “estrogen receptor signalling pathway” within SSEA‐1^+^ EECs in our *in silico* study suggest their restrained hormone responsiveness, in a monocellular organoid system that we employed, the SSEA‐1^+^ cells appear to respond to oestrogenic signals. However, the hormonal regulation of individual cell types in the intact endometrium is more complex, and the epithelial response to progesterone for example, is exerted via its action on the stromal cells [41]. Our organoid system did not contain stromal component, therefore was not suitable to fully assess the hormone regulation of SSEA1^+^ EECs that occur in vivo.

Differential expression analysis showed significantly higher expression of *VIM*, *PDGFRA*, and *PDGFRB* within the SSEA‐1^−^ EECs. This is likely to have been secondary to stromal contamination obtained from the flow‐through within the MACS cell separation process and is a recognized limitation of this technique. We have previously published the typical cell purity from MACs to be > 75% [2].

Our work has provided critical information regarding the transcriptional profile of premenopausal SSEA‐1^+^ EECs, supporting their pivotal role as progenitor cells driving endometrial regeneration and remodeling, which may play a role in protecting and regulating healthy endometrial homeostasis. Although SSEA‐1^+^ EECs generally reside in the basalis endometrial epithelium of healthy women, SSEA‐1^+^ EECs are also seen in the luminal epithelium [30] and in the functionalis of some women with gynecological pathologies such as endometriosis [10]. Further studies are now warranted using novel techniques such as spatial transcriptomics to assess in situ transcriptomics of different SSEA‐1^+^ EEC subpopulations. This would allow us to understand the functional differences of SSEA‐1^+^ EEC subtypes based on their region‐specific location within the endometrium. Further functional studies are required to explore SSEA‐1^+^ EEC mechanistic pathways in model systems that contain all main cell types recapitulating the in vivo endometrium and by manipulation of their upstream regulators.

## Author Contributions

D.K.H. conceived the project. H.A.‐L., A.V., O.V., J.D., C.J.H., and D.K.H. designed the experiments. H.A.‐L., J.D., C.J.H., and A.V. performed the experimental work. H.A.‐L., J.S., D.G., O.V., A.M., and N.T. were involved in the data analysis. H.A.‐L. and D.K.H. wrote the first draft of the manuscript. All authors, H.A.‐L., J.S., D.G., O.V., A.V., C.J.H., J.D., A.M., A.D., N.T., and D.K.H., were involved in the editing and approval of the final manuscript.

## Conflicts of Interest

University of Liverpool has received honoraria for consultancy from Theramex and has received payment for presentations from Theramex and Gideon Richter for D.K.H.'s work. The remaining authors have no Conflicts of interests to report.

## Supporting information


Figure S1.

Figure S2.

Figure S3.

Table S1.

Table S2.

Table S3.

Table S4.

Table S5.

Table S6.

## Data Availability

Raw and processed microarray datasets for the SSEA‐1 sorted EECs (SSEA‐1^+^ versus SSEA‐1^−^) were uploaded onto the Gene Expression Omnibus (GEO) at the National Center for Biotechnology Information (NCBI, http://www.ncbi.nlm.nih.gov/geo/) and can be accessed through accession number GSE280323. For external validation against previously published and publicly available endometrial single‐cell transcriptomic data, the data published by Garcia‐Alonso et al. [22] was downloaded. Processed endometrial epithelial matrices were accessed and downloaded from www.reproductivecellatlas.org. Datasets can also be accessed on ArrayExpress under accession number E‐MTAB‐10287 (scRNA‐seq in vivo) and the additional single‐cell transcriptomes published by Wang et al. of 10 endometrial biopsies from the Gene Expression Omnibus with GSE111976 [42]. Code used to perform the differential expression analysis is available at https://github.com/CBFLivUni/SSEA1_Microarray.

## References

[1] N. Tempest , C. J. Hill , A. Maclean , et al., “Novel Microarchitecture of Human Endometrial Glands: Implications in Endometrial Regeneration and Pathologies,” Human Reproduction Update 28 (2022): 153–171, 10.1093/humupd/dmab039. 34875046 PMC8888994

[2] A. J. Valentijn , K. Palial , H. Al‐Lamee , et al., “SSEA‐1 Isolates Human Endometrial Basal Glandular Epithelial Cells: Phenotypic and Functional Characterization and Implications in the Pathogenesis of Endometriosis,” Human Reproduction 28 (2013): 2695–2708, 10.1093/humrep/det285. 23847113

[3] R. W. S. Chan , K. E. Schwab , and C. E. Gargett , “Clonogenicity of Human Endometrial Epithelial and Stromal Cells1,” Biology of Reproduction 70 (2004): 1738–1750, 10.1095/biolreprod.103.024109. 14766732

[4] D. K. Hapangama , A. M. Kamal , and J. N. Bulmer , “Estrogen Receptor β: The Guardian of the Endometrium,” Human Reproduction Update 21 (2015): 174–193, 10.1093/humupd/dmu053. 25305176

[5] G. N. Papanicolaou , “Epithelial Regeneration in the Uterine Glands and on the Surface of the Uterus,” American Journal of Obstetrics and Gynecology 25 (1933): 30–37, 10.1016/S0002-9378(33)90417-2.

[6] V. A. Prianishnikov , “A Functional Model of the Structure of the Epithelium of Normal, Hyperplastic, and Malignant Human Endometrium: A Review,” Gynecologic Oncology 6 (1978): 420–428, 10.1016/0090-8258(78)90050-1. 367887

[7] F. Tresserra , P. Grases , A. Ubeda , M. A. Pascual , P. J. Grases , and R. Labastida , “Morphological Changes in Hysterectomies After Endometrial Ablation,” Human Reproduction 14 (1999): 1473–1477, 10.1093/humrep/14.6.1473. 10357962

[8] C. E. Gargett and D. Hapangama , “Endometrial Stem/Progenitor Cells: Prospects and Challenges,” Journal of Personalized Medicine 12 (2022): 1466, 10.3390/jpm12091466. 36143251 PMC9505339

[9] H. Al‐Lamee , C. J. Hill , F. Turner , et al., “The Role of Endometrial Stem/Progenitor Cells in Recurrent Reproductive Failure,” Journal of Personalized Medicine 12 (2022): 775, 10.3390/jpm12050775. 35629197 PMC9143189

[10] D. K. Hapangama , J. Drury , L. Da Silva , et al., “Abnormally Located SSEA1+/SOX9+ Endometrial Epithelial Cells With a Basalis‐Like Phenotype in the Eutopic Functionalis Layer May Play a Role in the Pathogenesis of Endometriosis,” Human Reproduction 34 (2019): 56–68, 10.1093/humrep/dey336. 30496412 PMC6295963

[11] N. Tempest , A. M. Baker , N. A. Wright , and D. K. Hapangama , “Does Human Endometrial LGR5 Gene Expression Suggest the Existence of Another Hormonally Regulated Epithelial Stem Cell Niche?,” Human Reproduction 33 (2018): 1052–1062, 10.1093/humrep/dey083. 29648645 PMC5972618

[12] H. P. T. Nguyen , C. N. Sprung , and C. E. Gargett , “Differential Expression of Wnt Signaling Molecules Between Pre‐ and Postmenopausal Endometrial Epithelial Cells Suggests a Population of Putative Epithelial Stem/Progenitor Cells Reside in the Basalis Layer,” Endocrinology 153 (2012): 2870–2883, 10.1210/en.2011-1839. 22474188 PMC3359601

[13] H. P. T. Nguyen , L. Xiao , J. A. Deane , et al., “N‐Cadherin Identifies Human Endometrial Epithelial Progenitor Cells by In Vitro Stem Cell Assays,” Human Reproduction 32 (2017): 2254–2268, 10.1093/humrep/dex289. 29040564

[14] M. E. Ritchie , J. Silver , A. Oshlack , et al., “A Comparison of Background Correction Methods for Two‐Colour Microarrays,” Bioinformatics 23 (2007): 2700–2707, 10.1093/bioinformatics/btm412. 17720982

[15] W. Huber , A. von Heydebreck , H. Sültmann , A. Poustka , and M. Vingron , “Variance Stabilization Applied to Microarray Data Calibration and to the Quantification of Differential Expression,” Bioinformatics 18, no. Suppl 1 (2002): S96–S104, 10.1093/bioinformatics/18.suppl_1.s96. 12169536

[16] Y. H. Yang , S. Dudoit , P. Luu , and T. P. Speed , “Normalization for cDNA Microarry dataMicroarrays: Optical Technologies and Informatics,” in Presented at the Microarrays: Optical Technologies and Informatics (SPIE, 2001), 141–152, 10.1117/12.427982.

[17] B. M. Bolstad , R. A. Irizarry , M. Astrand , and T. P. Speed , “A Comparison of Normalization Methods for High Density Oligonucleotide Array Data Based on Variance and Bias,” Bioinformatics 19 (2003): 185–193, 10.1093/bioinformatics/19.2.185. 12538238

[18] Y. Benjamini and Y. Hochberg , “Controlling the False Discovery Rate: A Practical and Powerful Approach to Multiple Testing,” Journal of the Royal Statistical Society: Series B (Methodological) 57, no. 1 (1995): 289–300, 10.1111/j.2517-6161.1995.tb02031.x.

[19] E. I. Boyle , S. Weng , J. Gollub , et al., “GO::TermFinder—Open Source Software for Accessing Gene Ontology Information and Finding Significantly Enriched Gene Ontology Terms Associated With a List of Genes,” Bioinformatics 20 (2004): 3710–3715, 10.1093/bioinformatics/bth456. 15297299 PMC3037731

[20] T. Wu , E. Hu , S. Xu , et al., “clusterProfiler 4.0: A Universal Enrichment Tool for Interpreting Omics Data,” Innovations 2 (2021): 100141, 10.1016/j.xinn.2021.100141. PMC8454663 34557778

[21] A. Krämer , J. Green , J. Pollard , and S. Tugendreich , “Causal Analysis Approaches in Ingenuity Pathway Analysis,” Bioinformatics 30, no. 4 (2014): 523–530, 10.1093/bioinformatics/btt703. 24336805 PMC3928520

[22] L. Garcia‐Alonso , L.‐F. Handfield , K. Roberts , et al., “Mapping the Temporal and Spatial Dynamics of the Human Endometrium In Vivo and In Vitro,” Nature Genetics 53 (2021): 1698–1711, 10.1038/s41588-021-00972-2. 34857954 PMC8648563

[23] Y. Hao , T. Stuart , M. H. Kowalski , et al., “Dictionary Learning for Integrative, Multimodal and Scalable Single‐Cell Analysis,” Nature Biotechnology 42 (2024): 293–304, 10.1038/s41587-023-01767-y. PMC10928517 37231261

[24] C. A. Schneider , W. S. Rasband , and K. W. Eliceiri , “NIH Image to ImageJ: 25 Years of Image Analysis,” Nature Methods 9 (2012): 671–675, 10.1038/nmeth.2089. 22930834 PMC5554542

[25] M. Y. Turco , L. Gardner , J. Hughes , et al., “Long‐Term, Hormone‐Responsive Organoid Cultures of Human Endometrium in a Chemically Defined Medium,” Nature Cell Biology 19, no. 5 (2017): 568–577, 10.1038/ncb3516. 28394884 PMC5410172

[26] I. S. Bostan , M. Mihaila , V. Roman , et al., “Landscape of Endometrial Cancer: Molecular Mechanisms, Biomarkers, and Target Therapy,” Cancers 16 (2024): 2027, 10.3390/cancers16112027. 38893147 PMC11171255

[27] S. A. Byron , M. Gartside , M. A. Powell , et al., “FGFR2 Point Mutations in 466 Endometrioid Endometrial Tumors: Relationship With MSI, KRAS, PIK3CA, CTNNB1 Mutations and Clinicopathological Features,” PLoS One 7 (2012): e30801, 10.1371/journal.pone.0030801. 22383975 PMC3285611

[28] K. C. Kurnit , G. N. Kim , B. M. Fellman , et al., “CTNNB1 (Beta‐Catenin) Mutation Identifies Low Grade, Early Stage Endometrial Cancer Patients at Increased Risk of Recurrence,” Modern Pathology 30 (2017): 1032–1041, 10.1038/modpathol. 28281553 PMC5493522

[29] D. K. Hapangama and J. N. Bulmer , “Pathophysiology of Heavy Menstrual Bleeding,” Womens Health 12 (2016): 3–13, 10.2217/whe.15.81. PMC5779569 26695831

[30] N. Tempest , A. Maclean , and D. K. Hapangama , “Endometrial Stem Cell Markers: Current Concepts and Unresolved Questions,” International Journal of Molecular Sciences 19, no. 10 (2018): 3240, 10.3390/ijms19103240. 30347708 PMC6214006

[31] A. I. Fraguas‐Sánchez , C. Martín‐Sabroso , and A. I. Torres‐Suárez , “Insights Into the Effects of the Endocannabinoid System in Cancer: A Review,” British Journal of Pharmacology 175 (2018): 2566–2580, 10.1111/bph.14331. 29663308 PMC6003657

[32] Q. Gao , F. Ye , X. Xia , et al., “Correlation Between PTEN Expression and PI3K/Akt Signal Pathway in Endometrial Carcinoma,” Journal of Huazhong University of Science and Technology. Medical Sciences 29 (2009): 59–63, 10.1007/s11596-009-0112-6. 19224164

[33] M.‐M. Georgescu , “PTEN Tumor Suppressor Network in PI3K‐Akt Pathway Control,” Genes & Cancer 1 (2010): 1170–1177, 10.1177/1947601911407325. 21779440 PMC3092286

[34] H. Lowe , N. Toyang , B. Steele , J. Bryant , and W. Ngwa , “The Endocannabinoid System: A Potential Target for the Treatment of Various Diseases,” International Journal of Molecular Sciences 22 (2021): 9472, 10.3390/ijms22179472. 34502379 PMC8430969

[35] A. M. Perevalova , V. S. Kobelev , V. G. Sisakyan , L. F. Gulyaeva , and V. O. Pustylnyak , “Role of Tumor Suppressor PTEN and Its Regulation in Malignant Transformation of Endometrium,” Biochemistry (Mosc) 87 (2022): 1310–1326, 10.1134/S0006297922110104. 36509719

[36] A. Kamal , N. Tempest , C. Parkes , et al., “Hormones and Endometrial Carcinogenesis,” Hormone Molecular Biology and Clinical Investigation 25 (2016): 129–148, 10.1515/hmbci-2016-0005. 26966933

[37] T. Kudo and H. Narimatsu , “Fucosyltransferase 3. GDP‐Fucose Lactosamine α1,3/4‐Fucosyltransferase. Lea and Leb Histo‐Blood Groups (FUT3, Lewis Enzyme),” in Handbook of Glycosyltransferases and Related Genes, ed. N. Taniguchi , K. Honke , M. Fukuda , H. Narimatsu , Y. Yamaguchi , and T. Angata (Springer, 2014), 531–539, 10.1007/978-4-431-54240-7_94.

[38] R. Nordén , K. Nyström , and S. Olofsson , “Activation of Host Antiviral RNA‐Sensing Factors Necessary for Herpes Simplex Virus Type 1‐Activated Transcription of Host Cell Fucosyltransferase Genes FUT3, FUT5, and FUT6 and Subsequent Expression of sLex in Virus‐Infected Cells,” Glycobiology 19 (2009): 776–788, 10.1093/glycob/cwp050. 19349624

[39] M. P. Caley , V. L. C. Martins , and E. A. O'Toole , “Metalloproteinases and Wound Healing,” Advances in Wound Care (New Rochelle) 4 (2015): 225–234, 10.1089/wound.2014.0581. PMC4397992 25945285

[40] R. Iglesias‐Bartolome , A. Uchiyama , A. A. Molinolo , et al., “Transcriptional Signature Primes Human Oral Mucosa for Rapid Wound Healing,” Science Translational Medicine 10, no. 451 (2018): eaap8798, 10.1126/scitranslmed.aap8798. 30045979 PMC6598699

[41] J. J. Kim , T. Kurita , and S. E. Bulun , “Progesterone Action in Endometrial Cancer, Endometriosis, Uterine Fibroids, and Breast Cancer,” Endocrine Reviews 34 (2013): 130–162, 10.1210/er.2012-1043. 23303565 PMC3565104

[42] W. Wang , F. Vilella , P. Alama , et al., “Single‐Cell Transcriptomic Atlas of the Human Endometrium During the Menstrual Cycle,” Nature Medicine 26 (2020): 1644–1653, 10.1038/s41591-020-1040-z. 32929266

