# Supplementary material for: Transcriptional Profiling of SSEA‐1+ Endometrial Epithelial Progenitor Cells Highlights Their Role in Endometrial Regeneration, Remodeling, and Homeostasis
Source: FASEB J. 2025 Apr 29;39(9):e70578. doi: 10.1096/fj.202402861R (PMC12038780; doi:10.1096/fj.202402861R)
Supplement: Supplementary file 1 — Figure S1. [file FSB2-39-e70578-s009.pdf]

Figure S1. *Representative canonical pathway predicted to be activated include* ***(A)*** *'PTEN signalling pathway'* ***(B)*** *'endocannabinoid cancer inhibition pathway'. Blue nodes represent genes/processed predicted to be inhibited. Orange nodes represent genes/processes predicted to be activated. Green nodes represent downregulated DEGs within the dataset. White nodes represent genes from IPA's Knowledge Base that are not part of the dataset. Purple Solid lines represent direct relationships (blue leading to inhibition and orange leading to activation). Dashed lines represent indirect relationships (blue leading to inhibition and orange leading to activation).*

A

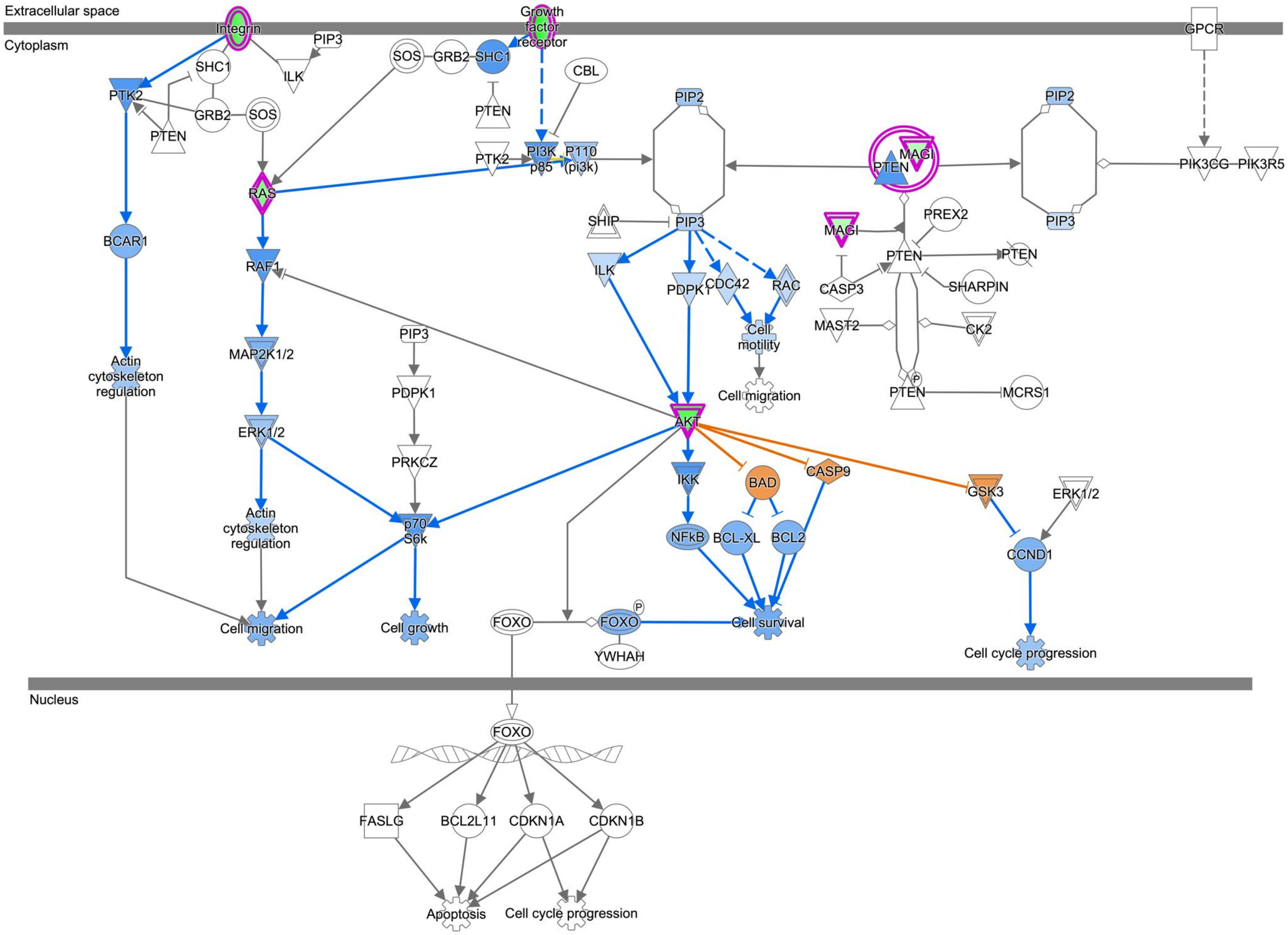
Extracellular space
Cytoplasm
Nucleus
Integrin
Growth factor receptor
GPCR
PIP3
SHC1
ILK
SOS
GRB2
CBL
PTK2
PTEN
PI3K p85
P110 (pi3k)
PIP2
MAGI
PIK3CG
PIK3R5
RAS
SHIP
PREX2
BCAR1
RAF1
CDC42
RAC
PDPK1
CASP3
SHARPIN
MAST2
CK2
Cell motility
Cell migration
MAP2K1/2
MCRS1
Actin cytoskeleton regulation
ERK1/2
PRKCZ
AKT
IKK
BAD
CASP9
GSK3
p70 S6k
NFkB
BCL-XL
BCL2
CCND1
Cell growth
FOXO
YWHAH
Cell survival
Cell cycle progression
FASLG
BCL2L11
CDKN1A
CDKN1B
Apoptosis

B

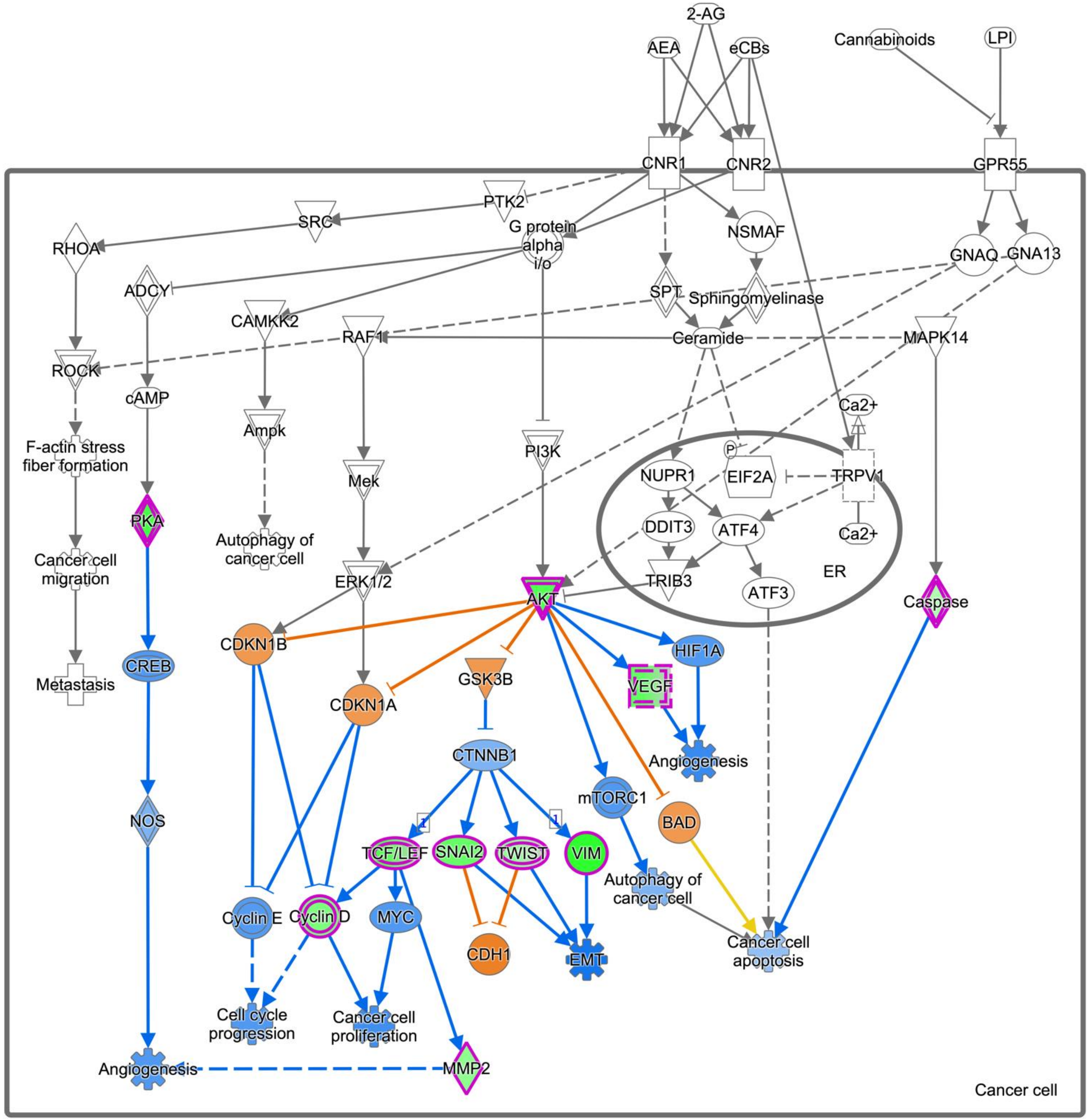
2-AG
AEA
eCBs
Cannabinoids
LPI
CNR1
CNR2
GPR55
PTK2
SRC
G protein alpha i/o
NSMAF
GNAQ
GNA13
RHOA
ADCY
CAMKK2
RAF1
SPT
Sphingomyelinase
Ceramide
MAPK14
ROCK
cAMP
Ampk
F-actin stress fiber formation
Mek
PI3K
Ca2+
P
NUPR1
EIF2A
TRPV1
DDIT3
ATF4
Ca2+
PKA
Autophagy of cancer cell
Cancer cell migration
ERK1/2
AKT
TRIB3
ATF3
ER
Caspase
CDKN1B
CREB
Metastasis
CDKN1A
GSK3B
HIF1A
VEGF
Angiogenesis
CTNNB1
mTORC1
BAD
NOS
TCF/LEF
SNAI2
TWIST
VIM
Autophagy of cancer cell
Cyclin E
Cyclin D
MYC
CDH1
EMT
Cancer cell apoptosis
Cell cycle progression
Cancer cell proliferation
Angiogenesis
MMP2
Cancer cell
