# Supplementary material for: Transcriptional Profiling of SSEA‐1+ Endometrial Epithelial Progenitor Cells Highlights Their Role in Endometrial Regeneration, Remodeling, and Homeostasis
Source: FASEB J. 2025 Apr 29;39(9):e70578. doi: 10.1096/fj.202402861R (PMC12038780; doi:10.1096/fj.202402861R)
Supplement: Supplementary file 2 — Figure S2. [file FSB2-39-e70578-s003.pdf]

Figure S2. *Canonical pathway 'estrogen receptor signalling' predicted to be inhibited. Orange nodes represent genes/processes predicted to be activated. Green nodes represent downregulated DEGs within the dataset. White nodes represent genes from IPA's Knowledge Base that are not part of the dataset. Purple Solid lines represent direct relationships (blue leading to inhibition and orange leading to activation). Dashed lines represent indirect relationships (blue leading to inhibition and orange leading to activation).*

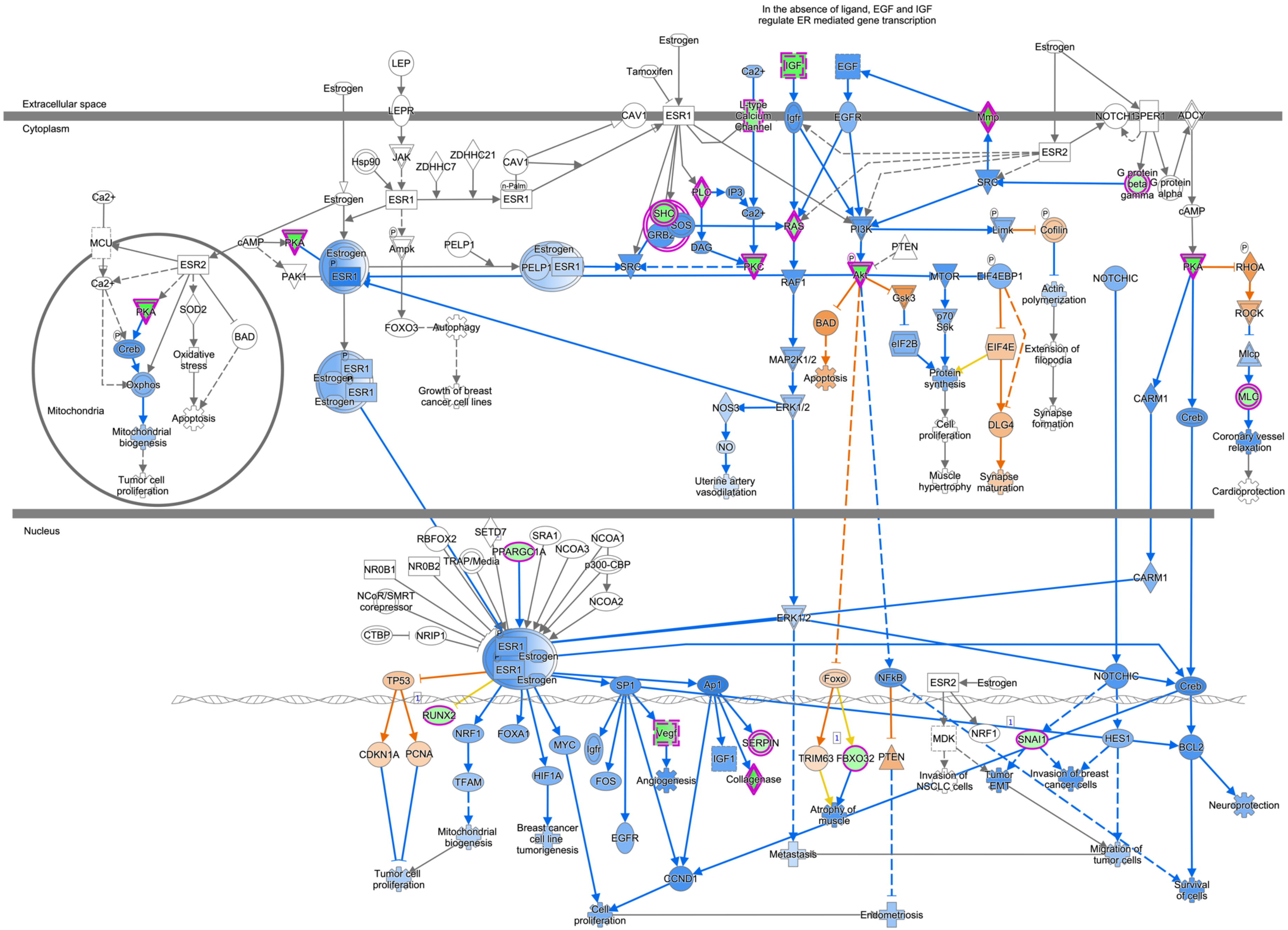
In the absence of ligand, EGF and IGF regulate ER mediated gene transcription
Extracellular space
Cytoplasm
Nucleus
Mitochondria
Estrogen
Tamoxifen
LEP
LEPR
JAK
Hsp90
ZDHHC7
ZDHHC21
CAV1
ESR1
ESR2
Ca2+
IGF
EGF
Igfr
EGFR
Mmp
L-type Calcium Channel
NOTCH1
GPER1
ADCY
SRC
G protein beta gamma
G protein alpha
cAMP
PKA
MCU
Creb
Oxphos
SOD2
Oxidative stress
BAD
Apoptosis
Mitochondrial biogenesis
Tumor cell proliferation
PAK1
Ampk
PELP1
FOXO3
Autophagy
Growth of breast cancer cell lines
SHC
GRB2
SOS
PLC
IP3
DAG
PKC
RAS
PI3K
RAF1
MAP2K1/2
ERK1/2
NOS3
NO
Uterine artery vasodilatation
Akt
PTEN
Gsk3
MTOR
p70 S6k
eIF2B
Protein synthesis
Cell proliferation
Muscle hypertrophy
EIF4EBP1
EIF4E
DLG4
Synapse maturation
Limk
Cofilin
Actin polymerization
Extension of filopodia
Synapse formation
NOTCHIC
RHOA
ROCK
Mlcp
MLC
Coronary vessel relaxation
Cardioprotection
CARM1
RBFOX2
SETD7
SRA1
NCOA1
NCOA3
PPARGC1A
TRAP/Media
p300-CBP
NR0B1
NR0B2
NCoR/SMRT corepressor
NCOA2
CTBP
NRIP1
TP53
RUNX2
CDKN1A
PCNA
NRF1
TFAM
FOXA1
MYC
HIF1A
Breast cancer cell line tumorigenesis
SP1
Ap1
Igfr
FOS
EGFR
Vegf
Angiogenesis
IGF1
SERPIN
Collagenase
CCND1
Foxo
TRIM63
FBXO32
Atrophy of muscle
NFkB
MDK
Invasion of NSCLC cells
Tumor EMT
SNAI1
Invasion of breast cancer cells
HES1
BCL2
Neuroprotection
Migration of tumor cells
Survival of cells
Metastasis
Endometriosis
