# Supplementary material for: Transcriptional Profiling of SSEA‐1+ Endometrial Epithelial Progenitor Cells Highlights Their Role in Endometrial Regeneration, Remodeling, and Homeostasis
Source: FASEB J. 2025 Apr 29;39(9):e70578. doi: 10.1096/fj.202402861R (PMC12038780; doi:10.1096/fj.202402861R)

Figure S3. *Venn diagram to show the overlap between* ***(A)*** *7 upregulated (logFC >1, p-value =0.0011) and* ***(B)*** *33 downregulated (logFC <1, p-value= 0.33) DEGs across Nguyen et al microarray expression data and the SSEA-1 EEC microarray expression data (Al-Lamee et al).*

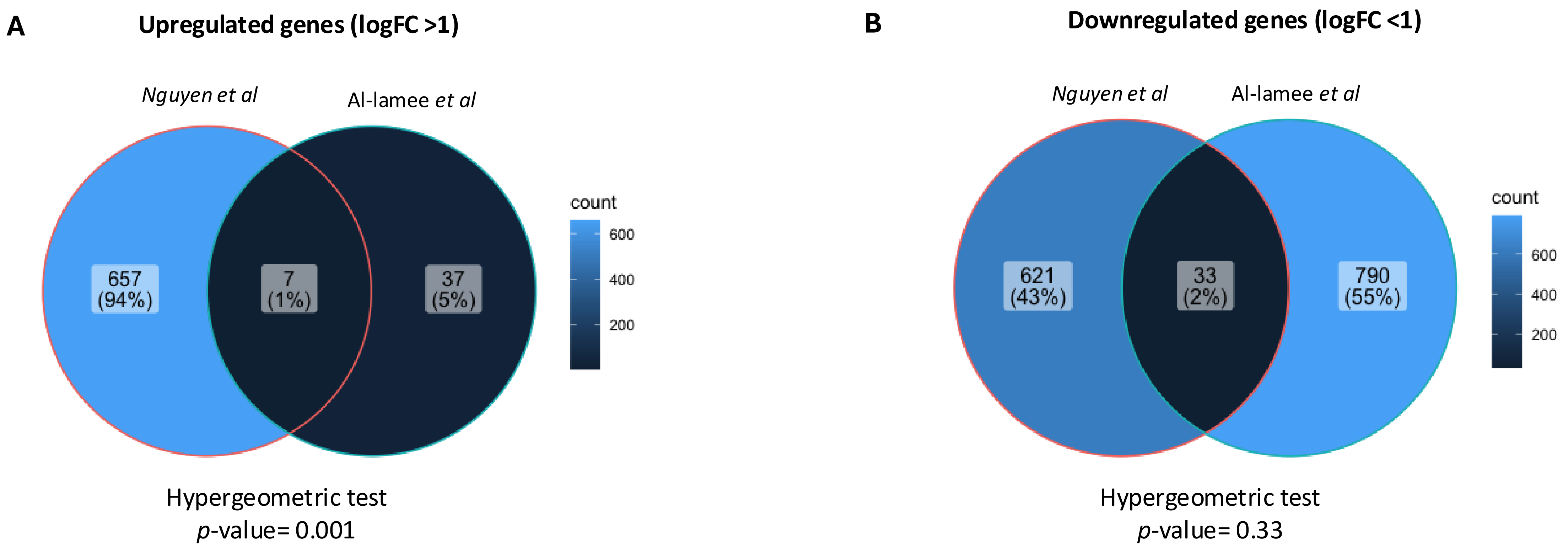

Supplement: Supplementary file 3 — Figure S3. [file FSB2-39-e70578-s007.pdf]
