# Supplementary material for: Transcriptional Profiling of SSEA‐1+ Endometrial Epithelial Progenitor Cells Highlights Their Role in Endometrial Regeneration, Remodeling, and Homeostasis
Source: FASEB J. 2025 Apr 29;39(9):e70578. doi: 10.1096/fj.202402861R (PMC12038780; doi:10.1096/fj.202402861R)
Supplement: Supplementary file 4 — Table S1. [file FSB2-39-e70578-s001.docx]

**Table S1.** Clinical characteristics of study participants (n=8). Full thickness samples were obtained from two patients (n=2) and pipelle samples were taken from the remaining six participants (n=6). Five samples were taken within the proliferative phase (n=5) and three within the secretory phase (n=3) of the cycle. Endometrial samples included in this table were used for microarray and RT-qPCR experiments. N51 excluded from further RT-qPCR analysis due to remaining RNA integrity.

| Study ID | Age (years) | BMI (kg/m^2^) | Smoker | Parity | Menstrual Cycle phase | Endometrial Sample Type |
| --- | --- | --- | --- | --- | --- | --- |
| N49 | 44 | 24.5 | No | 2 | Proliferative | Pipelle |
| N51 | 30 | 25 | No | 0 | Proliferative | Pipelle |
| N52 | 47 | 27.7 | Yes | 4 | Secretory | Full thickness |
| N53 | 40 | 32.4 | No | 2 | Secretory | Pipelle |
| N54 | 43 | 35.2 | No | 3 | Secretory | Pipelle |
| N55 | 32 | 27.8 | Yes | 2 | Proliferative | Full thickness |
| N58 | 31 | 39.8 | No | 3 | Proliferative | Pipelle |
| N59 | 42 | 29.8 | No | 2 | Proliferative | Pipelle |
