# Supplementary material for: Transcriptional Profiling of SSEA‐1+ Endometrial Epithelial Progenitor Cells Highlights Their Role in Endometrial Regeneration, Remodeling, and Homeostasis
Source: FASEB J. 2025 Apr 29;39(9):e70578. doi: 10.1096/fj.202402861R (PMC12038780; doi:10.1096/fj.202402861R)
Supplement: Supplementary file 5 — Table S2. [file FSB2-39-e70578-s008.docx]

**Table S2.** Primers used for RT-qPCR

| **Gene** | **Supplier** | **Amplicon context sequence/Primer sequence** | **Amplicon size (bp)** |
| --- | --- | --- | --- |
| ***Upregulated genes*** | |  |  |
| *MMP7* | Bio-Rad (qHsaCID0011537) | AATGAATGGATGTTCTGCCTGAAGTTTCTATTTCTTTCTTGAATTACTTCTCTTTCCATATAGTTTCTGAATGCCTTTAATATCATCCTGGGAAAGTTTAAAATTTTGGGGATCTCCATTTCCATAGGTTGGATACATCACTGCATTAGGACAGAGGAATGTCCCAT | 138 |
| *MMP26* | Bio-Rad (qHsaCID0016002) | AGACCATAAAGGATGGGACTTTGTTGAGGGCTATTTCCATCAATTTTTCCTGACCAAGAAGGAGTCGCCACTCCTTACCCAGGAGACACAAACACAGCTCCTGCAACAATTCCATCGGAATGGGACAGACCTACTTGACATGCAGATGCATGCTCTGCT | 129 |
| *SPRR2A* | Bio-Rad (qHsaCED0003548) | ATCATGGGCAGATTACTGGCTAAGGAGAAAGAAGCTCCCTGTGTATCCATGGTAGGCTTTGATGAGAAGATGAAGGTGGAGCTGTGGAACGAGGTGAGCCAAATATCCTTATCCTTTCTTGGTCCTGATGAATTCTGAAGCTGTTACTTGCTCTTCGGTGGATACTTTGACTGGCAGGGTGGGGAAGGTGTCACAGGAGGATATTTCTGCTGGCACTGCTGCTGAGGTG | 196 |
| *FUT3* | Sigma | F- GCCGACCGCAAGGTGTAC R- TGACTTAGGGTTGGACATGATATCC | 75 |
| ***Downregulated genes*** | |  |  |
| *ST3GAL2* | Bio-Rad (qHsaCID0016088) | TTCTTGGCACTCTCAGGGTACATGAAATGGTGGGTGGTTCGGCTGCCAACATCCTGCTCAAAGCCCACGGTTGGCGCCTGATTCATCCTCATGATGAAGTTGTGCCCGTCCACGTCCTGCCCATAGCCAGAG | 102 |
| *AXIN2* | Bio-Rad (qHsaCID0017930) | GGAGCACCGTCTCATCCTCCCAGATCTCCTCAAACACCGCTCCACAGGCAAACTCATCGCTTGCTTTTTTGAAGTAATACCTATAATTTCCCTTTTTGCTGAGCTGCTCTTTAAAGTGGCCCAGGGT | 97 |
| *TCF4* | Bio-Rad (qHsaCID0015722) | CCTGTCCTCCATTTCTAGACCAAACAGCTGTGCCTGCTGAGAGAGATGGAGGAGAGCCAACAGGAGTTGAAGGGTTTGATGAAAAGCTGTTGTTAGTGTGATCTGGAGAATAGATCGAAGCAAGTGCTTTCCCCA | 104 |
| *TMEM158* | Bio-Rad (qHsaCED0018889) | GCCAAAAAACCACATAAGTACAAGAAGAAACGCCCACCAGGCCACGTCACTGGATAGATCAACTTGGAAAAGGTTCATAAAGTTCATTTTCAGCGGTAACAATATATCGGTGTTAAATCCTTCCCATGCC CTCCCCCCAATTTTAAATAAA | 121 |
| *WNT5A* | Bio-Rad (qHsaCID0012240) | AGCCGGACACCCCATGGCACTTGCAGGCCACATCAGCCAGGTTGTACACCGTCCTGCGGCCGGCCTCGTTGTTGTGCAGGTTCATGAGGATGCGAGC | 67 |
| *ZEB1* | Bio-Rad (qHsaCED0045418) | GAAGATAACTTTAGTTGCTCCCTGTGCAGTTACACCTTTGCATACAGAACCCAACTTGAACGTCACATGACATCACATAAATCAGGAAGAGATCAAAG | 68 |
| ***Housekeeping genes*** | |  |  |
| *ACTB* | Sigma | F-TGTACGCCAACACAGTGCTG R-GCTGGAAGGTGGACAGCGA | 183 |
| *GAPDH* | Sigma | F- AATCCCATCACCATCTTCCA R- TGGACTCCACGACGTACTCA | 83 |
| *YWHAZ* | Sigma | F- CGTTACTTGGCTGAGGTTGCC R- GTATGCTTGTTGTGACTGATCGAC | 69 |
