# Supplementary material for: Transcriptional Profiling of SSEA‐1+ Endometrial Epithelial Progenitor Cells Highlights Their Role in Endometrial Regeneration, Remodeling, and Homeostasis
Source: FASEB J. 2025 Apr 29;39(9):e70578. doi: 10.1096/fj.202402861R (PMC12038780; doi:10.1096/fj.202402861R)
Supplement: Supplementary file 6 — Table S3. [file FSB2-39-e70578-s006.docx]

**Table S3*.*** Antibodies used for immunofluorescence

| **Antibody** | **Clone** | **Species** | **Unmasking buffer** | **Dilution** | **Supplier** | **Secondary** |
| --- | --- | --- | --- | --- | --- | --- |
| MMP7 | ab205525 | Rabbit | Citrate buffer | 1:500 | Abcam | Rabbit Alexa Fluor 555 |
| MMP26 | 13415-T10 | Rabbit | Citrate buffer | 1:500 | SinoBiological | Rabbit Alexa Fluor 555 |
| C11orf52 | 201636-T10 | Rabbit | Citrate buffer | 1:200 | SinoBiological | Rabbit Alexa Fluor 555 |
| CD47 | 12283-T26 | Rabbit | Citrate buffer | 1:1000 | SinoBiological | Rabbit Alexa Fluor 555 |
| Alexa Fluor 488 conjugated SSEA1 | MC-480/ 125610 | Mouse | Citrate buffer | 1:20 | BioLegend | - |
| Alexa Fluor 555 | #8953 | Rabbit | - | 1:1000 | Cell Signalling | - |
