# Supplementary material for: Transcriptional Profiling of SSEA‐1+ Endometrial Epithelial Progenitor Cells Highlights Their Role in Endometrial Regeneration, Remodeling, and Homeostasis
Source: FASEB J. 2025 Apr 29;39(9):e70578. doi: 10.1096/fj.202402861R (PMC12038780; doi:10.1096/fj.202402861R)
Supplement: Supplementary file 7 — Table S4. [file FSB2-39-e70578-s002.docx]

**Table S4.** Media components for organoid culture*.*

| **Component** | **Supplier** | **Final concentration** | **Expansion medium** | **Decidualisation medium** |
| --- | --- | --- | --- | --- |
| Phenol-free DMEM/F12 | Gibco™ | NA | **+** | **+** |
| N2 supplement | Life Technologies | 1X | **+** | **+** |
| B27 supplement | Life Technologies | 1X | **+** | **+** |
| Primocin | Invivogen | 100 μg/mL | **+** | **+** |
| L-glutamine | Sigma Aldrich | 2 mM | **+** | **+** |
| Human noggin | Peprotech | 100 ng/mL | **+** | **+** |
| Human EGF | Peprotech | 50 ng/mL | **+** | **+** |
| Human HGF | Peprotech | 50 ng/mL | **+** | **+** |
| Human FGF10 | Peprotech | 100 ng/mL | **+** | **+** |
| Human R-spondin-1 | Peprotech | 500 ng/mL | **+** | **+** |
| N-acetyl-L-cysteine | Sigma Aldrich | 1.25 mM | **+** | **+** |
| Nicotinamide | Sigma Aldrich | 10 nM | **+** | **+** |
| A83-01 | System Bioscience | 500 nM | **+** | **+** |
| Charcoal-stripped FBS | ThermoFisher Scientific | 10% [v/v] | **-** | **-** |
| E2 | Sigma Aldrich | 10 nM | **-** | **+** |
| MPA | Sigma Aldrich | 1 μM | **-** | **+** |
| cAMP | Sigma Aldrich | 500 μM | **-** | **+** |
