# Supplementary material for: Transcriptional Profiling of SSEA‐1+ Endometrial Epithelial Progenitor Cells Highlights Their Role in Endometrial Regeneration, Remodeling, and Homeostasis
Source: FASEB J. 2025 Apr 29;39(9):e70578. doi: 10.1096/fj.202402861R (PMC12038780; doi:10.1096/fj.202402861R)
Supplement: Supplementary file 8 — Table S5. [file FSB2-39-e70578-s005.docx]

**Table S5.** Antibodies used for immunohistochemistry

| **Antibody** | **Clone** | **Species** | **Unmasking buffer** | **Dilution** | **Supplier** |
| --- | --- | --- | --- | --- | --- |
| SSEA1 | MC 480 | Mouse | Tris EDTA | 1:800 | BioLegend |
| PR | PgR 636 | Mouse | Citrate buffer | 1:1000 | Dako |
| Ki67 | NCL-Ki67-MM1-L-CE | Mouse | Citrate buffer | 1:200 | Leica (Novocastra) |
| SOX9 | Polyclonal (AF3075) | Goat | Citrate buffer | 1:1000 | R&D Systems |
| N Cadherin | 8C11 | Rabbit | Tris EDTA | 1:100 | BioLegend |
