# Supplementary material for: Transcriptional Profiling of SSEA‐1+ Endometrial Epithelial Progenitor Cells Highlights Their Role in Endometrial Regeneration, Remodeling, and Homeostasis
Source: FASEB J. 2025 Apr 29;39(9):e70578. doi: 10.1096/fj.202402861R (PMC12038780; doi:10.1096/fj.202402861R)
Supplement: Supplementary file 9 — Table S6. [file FSB2-39-e70578-s004.docx]

**Table S6.** IPA downstream effects analysis table, showing the list of genes within the dataset that are predicted to influence the downstream predicted upregulated biofunction ‘organismal death’ (z-score= 14.60, FDR= 4.85e-32).

| **ID** | **Genes in dataset** | **Prediction (based on measurement direction)*** | **Expr Log Ratio** |
| --- | --- | --- | --- |
| NM_004751 | GCNT3 | Affected | 1.404 |
| NM_020890 | CIP2A | Affected | 1.274 |
| NM_003564 | TAGLN2 | Affected | 1.246 |
| NR_038940 | SH3PXD2A-AS1 | Affected | 1.182 |
| NM_002965 | S100A9 | Decreased | 1.17 |
| NM_003862 | FGF18 | Affected | 1.133 |
| NM_001657 | AREG | Affected | 1.117 |
| NM_005114 | HS3ST1 | Decreased | 1.088 |
| NM_198793 | CD47 | Decreased | 1.082 |
| NM_001562 | IL18 | Decreased | 1.076 |
| NM_206956 | PRAME | Affected | 1.065 |
| NM_002423 | MMP7 | Decreased | 1.063 |
| NM_005186 | CAPN1 | Decreased | 1.054 |
| NM_000594 | TNF | Increased | 1.05 |
| NM_181642 | SPINT1 | Decreased | 1.043 |
| NM_145699 | APOBEC3A | Affected | 1.008 |
| NM_004433 | ELF3 | Affected | 1.008 |
| NM_000492 | CFTR | Decreased | 1.001 |
| NM_001163 | APBA1 | Increased | -1 |
| NM_145040 | CAVIN3 | Increased | -1.004 |
| NM_004586 | RPS6KA3 | Affected | -1.006 |
| NM_024007 | EBF1 | Increased | -1.006 |
| NM_015656 | KIF26A | Increased | -1.008 |
| NM_006475 | POSTN | Increased | -1.011 |
| NM_022082 | SLC17A9 | Affected | -1.012 |
| NM_000358 | TGFBI | Increased | -1.013 |
| NM_005795 | CALCRL | Increased | -1.014 |
| XR_110214 | DSE | Affected | -1.014 |
| NM_025239 | PDCD1LG2 | Affected | -1.015 |
| NM_014899 | RHOBTB3 | Affected | -1.017 |
| NM_001452 | FOXF2 | Increased | -1.018 |
| NM_004385 | VCAN | Affected | -1.019 |
| NM_012242 | DKK1 | Increased | -1.02 |
| NM_005239 | ETS2 | Increased | -1.022 |
| NM_006426 | DPYSL4 | Affected | -1.024 |
| NM_000697 | ALOX12 | Increased | -1.026 |
| NM_006144 | GZMA | Increased | -1.026 |
| NM_033663 | DRD3 | Increased | -1.027 |
| NM_012301 | MAGI2 | Increased | -1.03 |
| NM_006439 | MAB21L2 | Increased | -1.032 |
| NM_003246 | THBS1 | Increased | -1.034 |
| NM_133263 | PPARGC1B | Increased | -1.038 |
| NM_005940 | MMP11 | Affected | -1.039 |
| NM_001099 | ACP3 | Affected | -1.041 |
| NM_004472 | FOXD1 | Affected | -1.045 |
| NM_015103 | PLXND1 | Increased | -1.049 |
| NM_000899 | KITLG | Increased | -1.051 |
| NM_014751 | MTSS1 | Increased | -1.052 |
| NM_002206 | ITGA7 | Increased | -1.053 |
| NM_016931 | NOX4 | Affected | -1.055 |
|  |  |  |  |

*Prediction (based on measurement direction): Affected = literature indicates this gene is involved in organismal death but does not indicate whether it increases or decreases it; increased = known in increase organismal death; decreased = known to decrease organismal death.
